# The promise(s) of mesenchymal stem cell therapy in averting preclinical diabetes: lessons from in vivo and in vitro model systems

**DOI:** 10.1038/s41598-021-96121-0

**Published:** 2021-08-20

**Authors:** Nagasuryaprasad Kotikalapudi, Samuel Joshua Pragasam Sampath, Sinha Sukesh Narayan, Bhonde R., Harishankar Nemani, Sathish Kumar Mungamuri, Vijayalakshmi Venkatesan

**Affiliations:** 1 Division of Cell and Molecular Biology, ICMR-National Institute of Nutrition, Jamai-Osmania P.O., Tarnaka, Hyderabad, 500007 India; 2 Division of Food Safety, ICMR-National Institute of Nutrition, Jamai-Osmania P.O., Tarnaka, Hyderabad, 500007 India; 3 Department of Regenerative Medicine, Manipal Institute of Regenerative Medicine, GKVK Post, Bellary Road, Allalasandra, Yelahanka, Bangalore, 560065 India; 4 Division of Animal Facility, ICMR-National Institute of Nutrition, Jamai-Osmania P.O., Tarnaka, Hyderabad, 500007 India; 5 Present Address: Dr. D. Y. Patil Vidyapeeth, Pune, 411018 India

**Keywords:** Mesenchymal stem cells, Cytokines, Stem cells, Insulin signalling, Metabolomics

## Abstract

Obesity (Ob) poses a significant risk factor for the onset of metabolic syndrome with associated complications, wherein the Mesenchymal Stem Cell (MSC) therapy shows pre-clinical success. Here, we explore the therapeutic applications of human Placental MSCs (P-MSCs) to address Ob-associated Insulin Resistance (IR) and its complications. In the present study, we show that intramuscular injection of P-MSCs homed more towards the visceral site, restored HOMA-IR and glucose homeostasis in the WNIN/GR-Ob (Ob-T2D) rats. P-MSC therapy was effective in re-establishing the dysregulated cytokines. We report that the P-MSCs activates PI3K-Akt signaling and regulates the Glut4-dependant glucose uptake and its utilization in WNIN/GR-Ob (Ob-T2D) rats compared to its control. Our data reinstates P-MSC treatment's potent application to alleviate IR and restores peripheral blood glucose clearance evidenced in stromal vascular fraction (SVF) derived from white adipose tissue (WAT) of the WNIN/GR-Ob rats. Gaining insights, we show the activation of the PI3K-Akt pathway by P-MSCs both in vivo and in vitro (palmitate primed 3T3-L1 cells) to restore the insulin sensitivity dysregulated adipocytes. Our findings suggest a potent application of P-MSCs in  pre-clinical/Ob-T2D management.

## Introduction

Obesity presenting with insulin resistance (IR), impaired glucose tolerance (IGT), and hyperglyceridemia (HG) has posed as a significant public health concern linked to several comorbidities like cardiovascular diseases, hypertension, chronic kidney disease, Alzheimer's disease, and certain cancers^1,2^. There has been an exponential increase in these associated metabolic pathologies, posing severe health threats worldwide, affecting over 40 to 50% of the adult population, because of which ~ 2.8 million people lose their lives globally every year^3,4^. The critical determinant underlying this pathophysiological state attributes to the inflammatory milieu (enlarged adipose tissue and macrophage infiltration) with sustained release of pro-inflammatory cytokines (TNFα, IL-1β, and IL-6)^5,6^, In addition, acute-phase cytokines and altered inflammatory signaling pathways in adipose tissue form a linking factor for several metabolic syndromes like Obesity and Type 2 Diabetes (Ob-T2D)^2^. Adipose tissue is the major contributor for the circulating IL-1β, TNFα, IL-6 during obese conditions, wherein IL-1β receptors are shown to induce major inflammatory signaling kinases and transcriptional pathways that result in impaired insulin receptor functions and altered Glut4 translocation in adipose tissue^5,6^. In obese people, the risk of T2D is primarily determined by the site of fat accumulation^7–9^. Adipose tissue, skeletal muscle, liver, and brain to a lesser extent, etc., are the significant depots of fat accumulation. Excess free fatty acids (FFA) in the obese condition contribute to the IR observed in these target tissues resulting in altered energy homeostasis^10^. Obesity has been associated with high infiltration and accumulation of macrophages within the adipose tissue^11^, and it is of utmost interest to note that macrophages can induce and secrete a range of pro-inflammatory cytokines that cause tissue inflammation and trigger the secretion of other pro-inflammatory molecules^12^.

Current therapies widely being used for Ob-T2D includes metformin, wherein it lowers liver glucose production^13,14^, besides its beneficial role in improving insulin sensitivity in the skeletal muscle^15,16^, and in the at WAT^17,18^. The other approved drugs for T2D include DPP-4 inhibitors, Sulfonylureas, and Meglitinides, whose treatment generally increases insulin secretion and/or glucose utilization^19,20^, in addition to exogenous insulin application to effectively ameliorate hyperglycemia and also improve the insulin response in target tissues, but owes limitations of short-term duration. Recent advances in identifying stem cells, more so adult stem cells/mesenchymal stem cells (MSCs) have gained attention in regenerative research due to their inherent immunomodulatory, multilineage, self-renewal, and potent secretome functions. MSCs modulate their cellular microenvironment, activate endogenous/resident progenitor cells, and secrete various factors^21^, and appear to hold promise(s) to improve healthcare by either augmenting body's own regenerative potential, such as in wound healing, ischemic diseases, neurological disorders, and diabetes mellitus, or development of new therapies^22^. The therapeutic effects of MSCs have been reported in diabetic subjects, diabetic models^23^ showing that MSCs could lower blood glucose levels. Other strategies to promote β-cell regeneration/repopulation vis-à-vis overcome IR in the target tissues (muscle/adipose) also open up alternate options for the management of T2D. Despite several options, the precise mechanism(s) underlying these effects are still obscure.

The impetus obtained from our earlier studies^24,25^, and other related studies portrays Ob-T2D as a state of profound stress and inflammation^5,6^. Interestingly, immunomodulatory effects of the human placental mesenchymal stem cells (P-MSCs) to blunt inflammatory response and allow tissue remodeling after injury^26,27^ reinstate their efficacy for safe transplantation. Recently, the promise(s) of Wharton's Jelly-derived MSCs to differentiate into islet-like clusters (ILCs)^28^, P-MSCs into ILCs^22^ have renewed interest for several β-cell researchers. The efficacy and feasibility of MSC therapy in obesity-induced pre-clinical diabetic conditions, interfacing with the onset of chronic diseases, have not been explored to date.

In the present study, we aim to explore the possible therapeutic efficacy of P-MSCs using WNIN/GR-Ob (Ob-T2D) rats as they portray both obesity & diabetic-related phenotype with IR^24–27^, developed indigenously at the National Center for Laboratory Animals Science (NCLAS) facility of ICMR-National Institute of Nutrition (NIN). They also show accelerated aging with a reduced average life span being 1.5 years compared to 2.5–3 years seen in the controls^24–27^. In the present study, we report for the first time the beneficial effects of P-MSCs treatment in WNIN/GR-Ob (Ob-T2D) mutant rats, depicting pre-clinical/clinical T2D and presents with frank obesity, IR, lean body mass, higher fat mass, hyper-insulinemia, hyper-triglyceridemia, hyper-cholesterolemia, and hyper-leptinemia, akin to human subjects of obesity/obesity-induced T2D. To pin down the mechanistic insights of P-MSCs in situ, we carried out in vitro experiments with adipocytes derived from palmitate primed 3T3-L1 cells and co-cultured with and without P-MSCs to assess the beneficial effects of P-MSCs.

We hypothesize that intramuscular injections of P-MSCs to WNIN/GR-Ob (Ob-T2D) rats with IR would ameliorate the obesogenic milieu in adipose tissue and counter the IR. These injections are likely to modulate the microenvironment/niche of adipose tissue to modulate adipokine secretions so as to blunt inflammation vis-à-vis enhance glucose sensitivity via Glut4 translocation to overcome IR. To test our hypothesis, we aim to explore the possible therapeutic efficacy of P-MSCs using WNIN/GR-Ob (Ob-T2D) rats studied in both subcutaneous and visceral depots and compare them with WNIN/Controls to evaluate) glycaemic status (IGTT, ITT, HOMA-IR), cytokine profiling, insulin signaling, metabolites, and Glut4 transportation and translocation.

## Results

### In vitro characterization of P-MSCs

**Figure 1:**
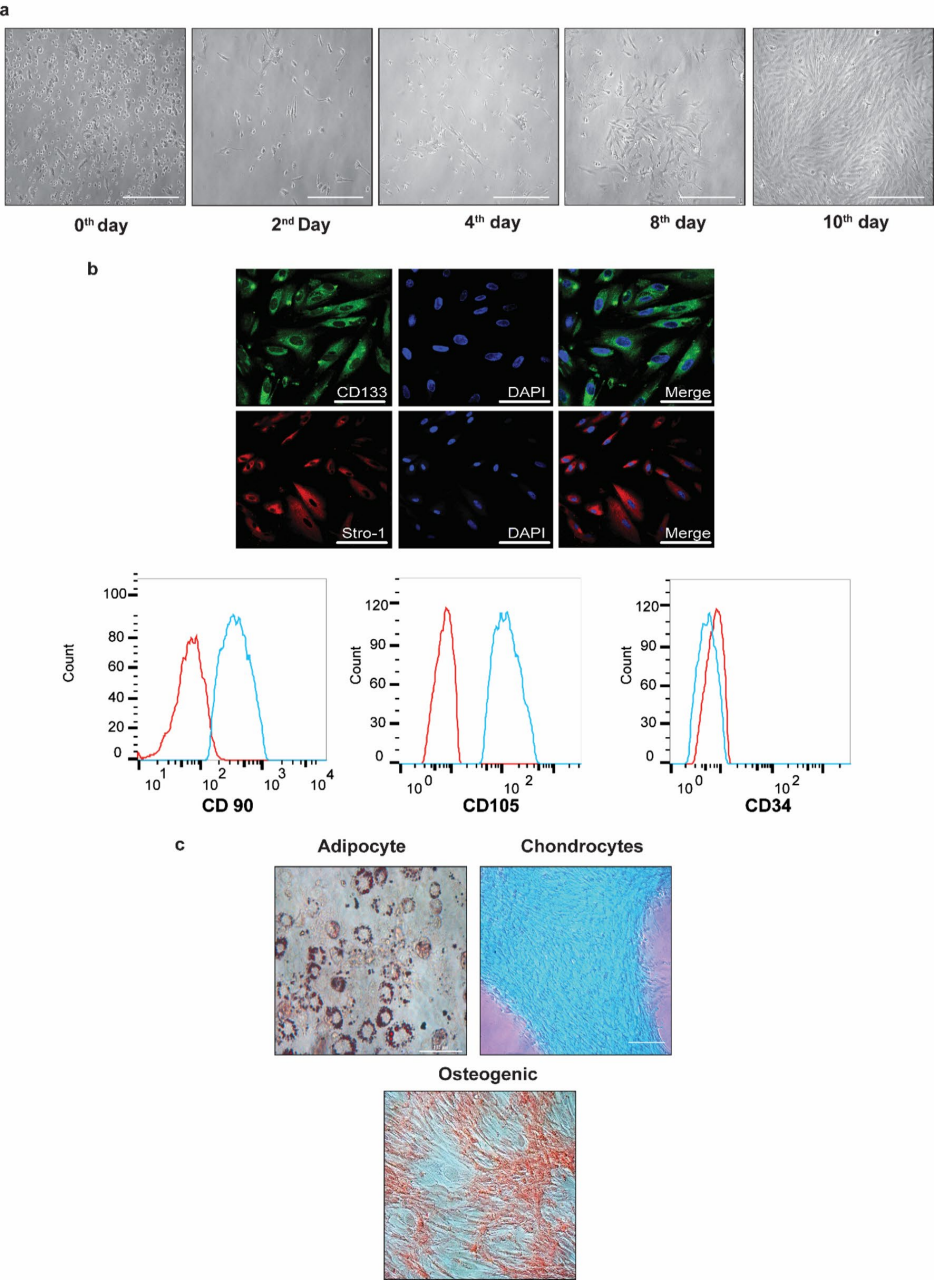
Morphology of placental-derived mesenchymal stem cells in primary cultures. (**a**) Bright-field images taken at the indicated time points showing the heterogeneous population's formation to a homogenous population of human placental mesenchymal stem cells (P-MSCs). (**b**) Representative confocal images of human P-MSCs showing positive staining for CD133 and Stro-1 stem cell markers. The nuclei were stained with DAPI, and a representative flow cytometer histogram of P-MSC for markers CD90, CD105, and CD34. (**c**) Representative microscopic images showing the differentiation of P-MSCs under various growth conditions. The presence of lipid droplets indicates adipogenic differentiation. Chondrogenic differentiation was demonstrated by the formation of Alcian blue positive pellets. Alizarin Red detected calcium depositions to show osteogenic differentiation.

Human P-MSCs were isolated from the placenta's chorionic side, which was highly proliferative, and showed colony-forming units in the bright field microscope (Fig. 1a). These cells were positive for CD133, STRO-1, CD90, and CD105 and were negative for CD34. P-MSCs demonstrated multilineage capacity and differentiated into adipogenic, chondrogenic, and osteogenic lineages, when cultured in appropriate differentiation conditions. These results support their mesenchymal phenotype (Fig. 1b,c).

### P-MSCs home to the visceral region in WNIN/GR-Ob (Ob-T2D) rats

**Figure 2:**
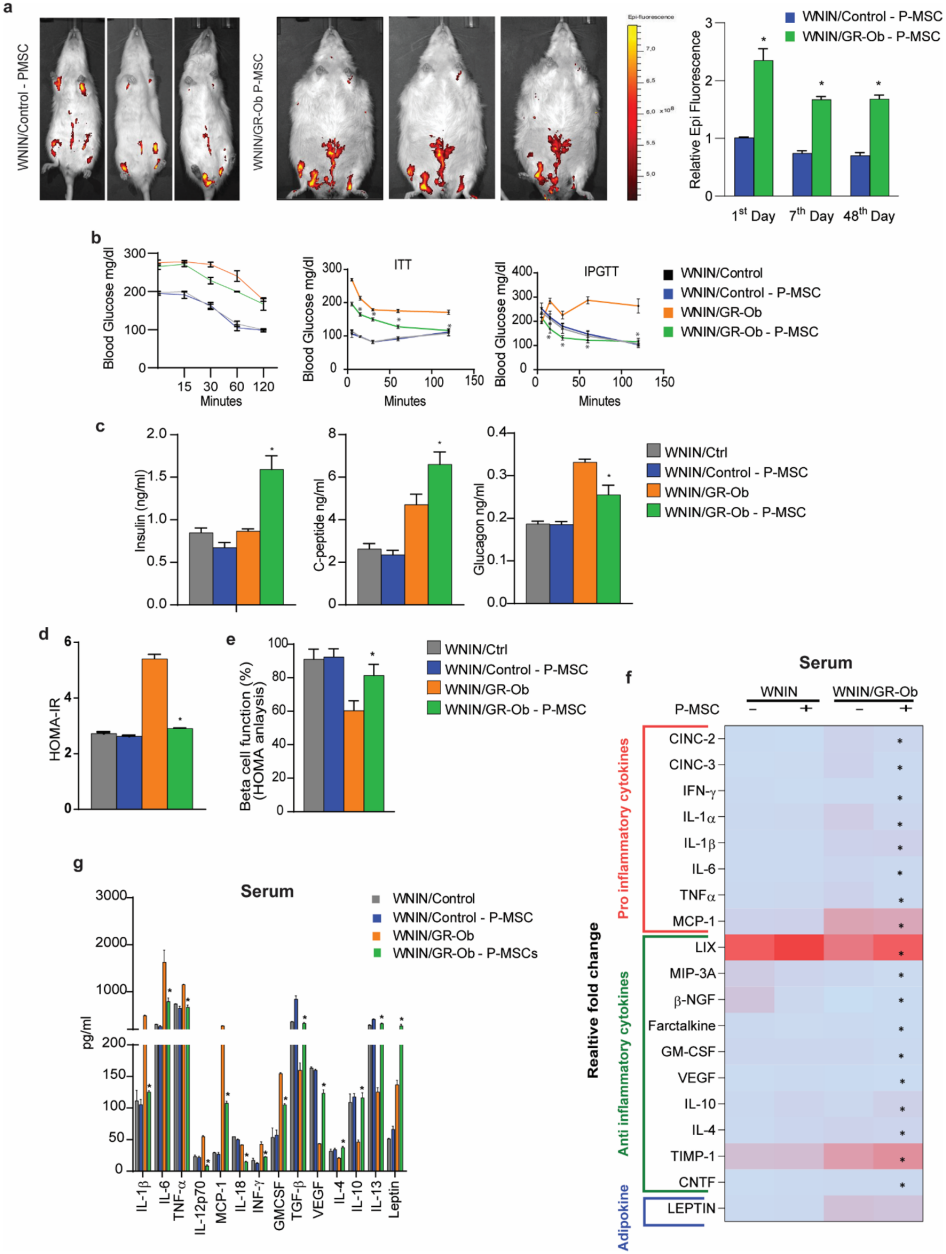
P-MSCs ameliorate hyperglycemia and insulin sensitivity in obesity-induced Type 2 diabetes complication in WNIN/GR-Ob (Ob-T2D) rats. (**a**) In vivo imaging showing the tracking of DiD labeled P-MSCs after intramuscular injection into WNIN/Control and WNIN/GR-Ob (Ob-T2D) rats on 1, 7, and 48 days. Note that warmer colors indicate more accumulation of cells. A bar diagram indicating the relative epifluorescence in each of the condition is also shown next to the in vivo imaging. The data is normalized to the epifluorescence taken at day 1 of WNIN/Control rats injected with P-MSCs. (**b**) Line diagram showing the glucose concentration in WNIN/Control and WNIN/GR-Ob (Ob-T2D) rats after a glucose tolerance test (intraperitoneal glucose tolerance test [IPGTT], before and after P-MSCs injection and insulin tolerance test (intraperitoneal insulin tolerance test [ITT]), before and after P-MSCs injection. (**c**) Bar diagram indicating the levels of Insulin, C-peptide, Glucagon in WNIN/Control and WNIN/GR-Ob (Ob-T2D) rats with and without P-MSCs injection. (**d**,**e**) Bar diagram indicating the levels of HOMA-IR and levels of HOMA-β in WNIN/Control and WNIN/GR-Ob (Ob-T2D) rats, with and without P-MSCs injection. (**f**) Heat maps showing the cytokine relative fold change in the serum of WNIN/Control and WNIN/GR-Ob (Ob-T2D) rats, with and without human P-MSCs injection. Colours are assigned according to the relative scale of expression, ranging from 0–150 au (arbitrary units) for serum, representing a fold-increase change in WNIN/GR-Ob (Ob-T2D) rats vs. WNIN/Control rats. (**g**) Pro- and anti-inflammatory cytokine concentration profile in serum in WNIN/Control and WNIN/GR-Ob (Ob-T2D) rats, with and without human P-MSCs injection, using Luminex system**.** Statistical analyses were performed using two-way ANOVA comparing the Control group and the P-MSCs injected group of WNIN/Control and WNIN/GR-Ob (Ob-T2D) rats (*p < 0.05, **p < 0.01). The error bars represent one standard deviation from the mean. n = 6 rats per group.

We next studied the homing capacity of P-MSCs to assess their migration in WNIN/Control and WNIN/GR-Ob (Ob-T2D) rats. We intramuscularly injected the P-MSCs (1 × 10^6^ in 100 µl 1× PBS), that were labeled with DiD (DilC_18_(5) solid (1,1′-Dioctadecyl-3,3,3′,3′-Tetramethylindodicarbocyanine, 4-Chlorobenzenesulfonate Salt). We followed its epifluorescence in a time-course manner (24 h to 7 weeks at regular intervals) using in vivo imaging. Our results show that the injected P-MSCs homed primarily to the visceral region in WNIN/GR-Ob (Ob-T2D) rats as compared to the WNIN/Control rats (Fig. 2a).

### P-MSCs treatment clears peripheral blood glucose and restores insulin utilization in WNIN/GR-Ob (Ob-T2D) rats

To understand the beneficial effect of P-MSCs in clearing the peripheral blood glucose and restoration of insulin utilization in Ob-T2D conditions, we performed the ITT and GTT assays in WNIN/GR-Ob (Ob-T2D) rats, with and without P-MSCs injection, and compared it with the WNIN/Control rats. As shown previously^29^, the WNIN/GR-Ob (Ob-T2D) rats show decreased glucose tolerance compared to the WNIN/Control rats (Fig. 2b). GTT and ITT assays using the peripheral blood indicate that treatment with P-MSCs improves the glucose utilization in WNIN/GR-Ob (Ob-T2D) rats compared to the WNIN/Control rats (Fig. 2b). Besides, we also observed that the WNIN/GR-Ob (Ob-T2D) rats, in response to P-MSCs injection, showed a significant increase in the serum insulin and C-peptide levels and a considerable decrease in the serum glucagon level (Fig. 2c). Further, we also observed decreased lipid peroxidation and reduced total cholesterol, total triglycerides, LDL, HDL, VLDL levels in the serum isolated from the WNIN/GR-Ob (Ob-T2D) rats in response to P-MSCs treatment (Supplementary Fig. 1a). “HOMA-IR” analysis is a widely accepted methodology for measuring IR and β-cell function^30^. Using this approach, previously, it was shown that WNIN/GR-Ob (Ob-T2D) rats have nearly twofold higher IR, with a twofold reduction in β-cell function^24,25,29^. Significantly, P-MSCs treatment reduced the IR in WNIN/GR-Ob (Ob-T2D) rats and enhanced the β-cell function to a level comparable to that of WNIN/Control rats (Fig. 2d,e). Our data confirm that de novo, P-MSCs injection increases insulin sensitivity and enhances glucose utilization in WNIN/GR-Ob (Ob-T2D) rats.

### P-MSCs treatment restores the cytokine expression of the WNIN/GR-Ob (Ob-T2D) rats

**Figure 3:**
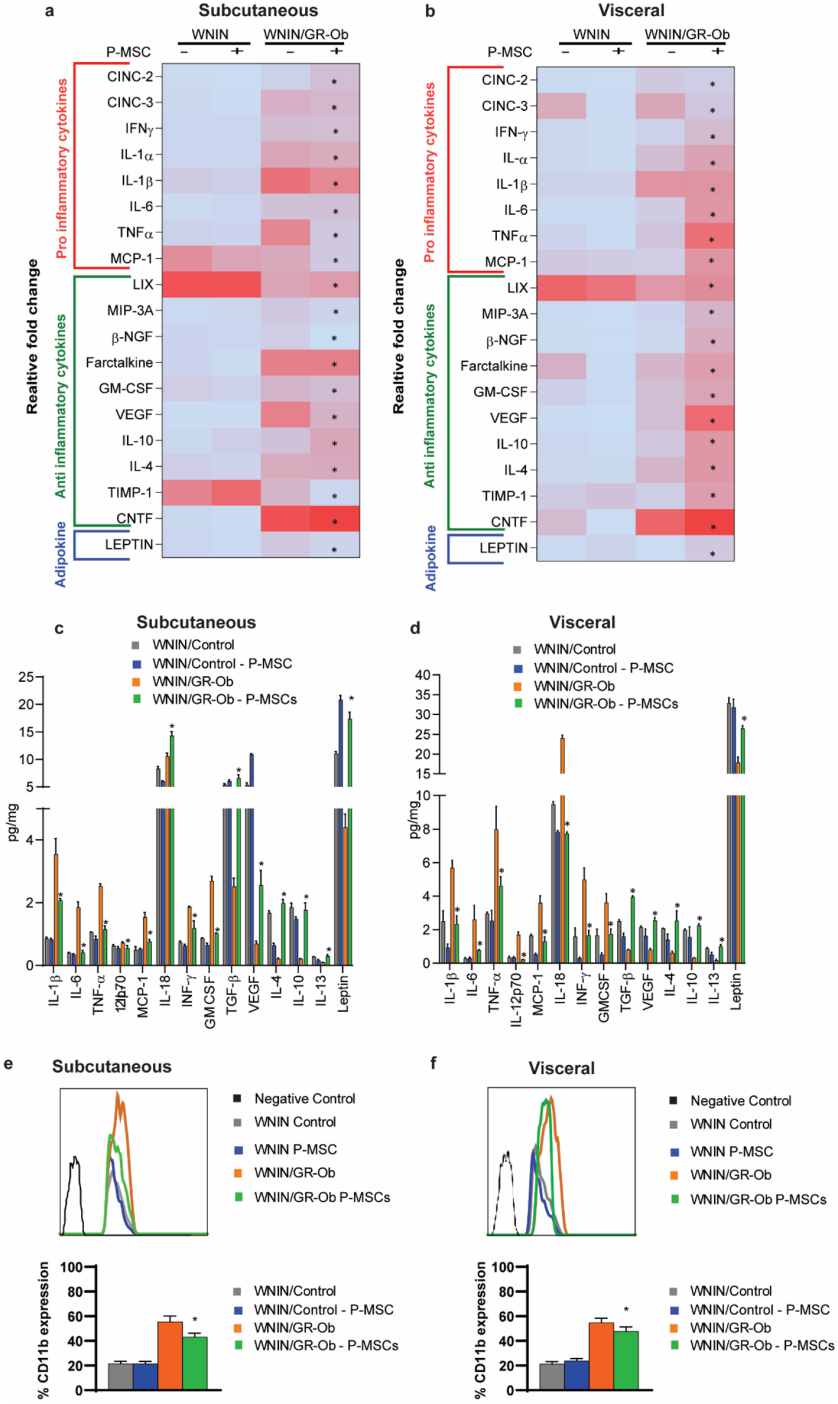
Human P-MSCs treatment restores the cytokine expression. Heat maps showing the cytokine concentrations in the subcutaneous (**a**), visceral (**b**) adipose tissues in WNIN/Control, and WNIN/GR-Ob (Ob-T2D) rats, with and without human P-MSCs injection. Colours are assigned according to the relative scale of expression, ranging from 0 to 40 au for adipose tissue and representing a fold-increase change in WNIN/GR-Ob (Ob-T2D) rats compared to WNIN/Control rats. (**c**,**d**) Pro- and anti-inflammatory cytokine concentration profile in subcutaneous (**c**), visceral (**d**) adipose tissues in WNIN/Control and WNIN/GR-Ob (Ob-T2D) rats, with and without human P-MSCs injection, using Luminex system. FACS analysis showing the expression of CD11b positive cells in the SVF fraction of subcutaneous (**e**) and visceral (**f**) adipose tissues of WNIN/Control and WNIN/GR-Ob (Ob-T2D) rats with or without P-MSCs injection. A bar diagram indicating the % of CD11b positive cells in each of the condition is also shown next to the FACS profiles. Statistical analyses were performed using two-way ANOVA comparing the control group and the P-MSCs injected group of WNIN/Control and WNIN/GR-Ob (Ob-T2D) rats (*p < 0.05, **p < 0.01). The error bars represent one standard deviation from the mean. n = 6 rats per group.

Next, we addressed whether the P-MSCs injection also leads to cytokine remodeling. Using the dot blot assay, we analyzed both pro-and anti-inflammatory cytokine expression in the serum (Fig. 2f, Supplementary Fig. 2a) and in subcutaneous adipose (Fig. 3a, Supplementary Fig. 2b) and visceral adipose tissues (Fig. 3b, Supplementary Fig. 2c), from WNIN/Control and WNIN/GR-Ob (Ob-T2D) rats, with and without P-MSCs injection. WNIN/GR-Ob (Ob-T2D) rats showed an increased expression of pro-inflammatory cytokines IL-1α, IL-1β, IFN-γ, IL-6, MCP-1, MIP-3A, CINC-2, CINC-3, GM-CSF, and TNF-α in their serum and adipocyte milieu, when compared to the WNIN/Control rats (Figs. 2f, 3a,b; Supplementary Fig. 2a–c). Similarly, WNIN/GR-Ob (Ob-T2D) rats had a decreased expression of anti-inflammatory cytokines IL-10, IL-4, VEGF, β-NGF, Fractalkine, CNTF, LIX, and TIMP-1 in their adipocyte milieu and in their serum, when compared to the WNIN/Control rats (Figs. 2f, 3a,b; Supplementary Fig. 2a–c). Leptin is a well-established adipokine and regulates the food intake capacity^31,32^. In addition to the alterations in the cytokine profile, we also observed that WNIN/GR-Ob (Ob-T2D) rats have decreased Leptin levels in their adipose milieu as well as in their serum when compared to the WNIN/Control rats (Figs. 2f, 3a,b; Supplementary Fig. 2a–c).

P-MSCs injection into the WNIN/Control rats had no significant impact on the cytokine expression levels (Figs. 2f,g, 3a–d; Supplementary Fig. 2a–c). Interestingly, we observed that P-MSCs injection to WNIN/GR-Ob (Ob-T2D) rats had restored the expression of both the pro-and anti-inflammatory cytokines, as well as Leptin expression, to the extent seen in the WNIN/Control rats (Figs. 2f,g, 3a,b; Supplementary Fig. 2a–c). We also reconfirmed the above observations that the P-MSCs injection restores cytokine profile in the WNIN/GR-Ob (Ob-T2D) rats, as shown by Multiplex-ELISA (Figs. 2g, 3c,d). Our qRT-PCR analysis gave a similar cytokine expression pattern at their respective mRNA levels, suggesting that the restoration of these cytokine alterations occurs at the transcriptional level (Supplementary Fig. 2a,b). These data confirm that P-MSCs injection re-establishes the normal cytokine profile in the WNIN/GR-Ob (Ob-T2D) rats.

### P-MSCs treatment reduces the macrophage infiltration into adipose tissues of the WNIN/GR-Ob (Ob-T2D) rats

We next analyzed the extent of macrophage infiltration, since there was a reversal of cytokine expression with P-MSCs therapy in the WNIN/GR-Ob (Ob-T2D) rats. FACS and H&E analysis showed that both subcutaneous and visceral adipose tissues of the WNIN/GR-Ob (Ob-T2D) rats are enriched with CD11b+ macrophages compared to WNIN/Control WAT tissues (Fig. 3e,f, and Supplementary Fig. 3). P-MSCs injection had substantially lessened the macrophage infiltration by more than 1.4-fold in the WAT of WNIN/GR-Ob (Ob-T2D) rats (Fig. 3e,f).

### P-MSCs treatment restores glucose homeostasis in the adipose tissues of WNIN/GR-Ob (Ob-T2D) rats

**Figure 4:**
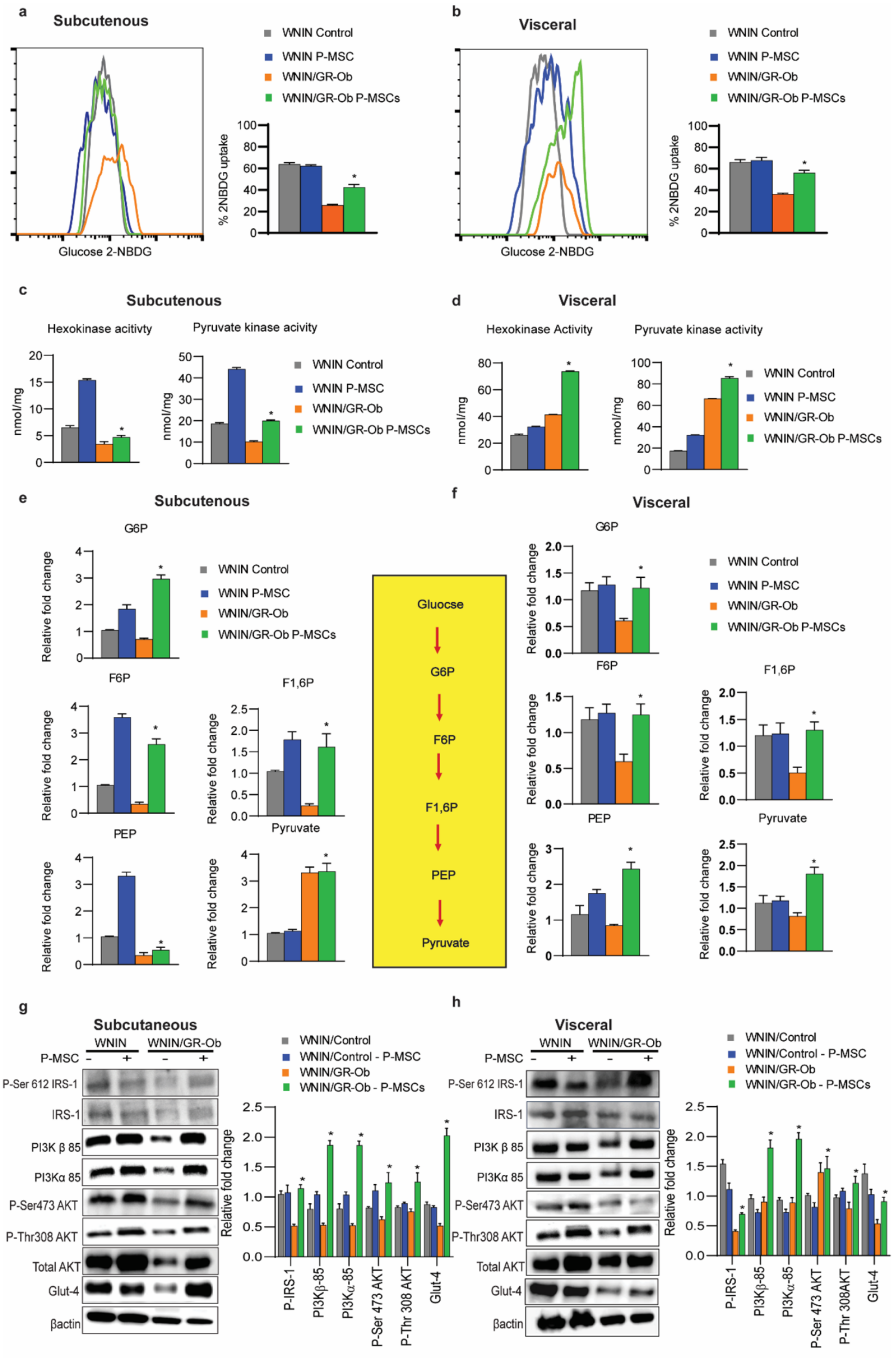
Human P-MSCs therapy restores glucose homeostasis in WNIN/GR-Ob (Ob-T2D) rats in subcutaneous and visceral adipose tissues. P-MSCs treatment restores PI3K-Akt signaling and Glut4 expression in the WNIN/GR-Ob (Ob-T2D) rats adipose tissues. (**a**,**b**) FACS analysis showing the relative 2-NBDG uptake in subcutaneous (**a**) and visceral (**b**) adipose tissues from WNIN/Control and WNIN/GR-Ob (Ob-T2D) rats treated with P-MSCs in the absence and presence of insulin stimulation. Data were normalized to the PBS group in the basal condition. The % 2-NBDG uptake in each of the condition is also shown in the form of bar diagram. (**c**,**d**) Bar diagrams showing the activities of Hexokinase and Pyruvate Kinase in subcutaneous (**c**) and visceral (**d**) adipose tissues of WNIN/Control and WNIN/GR-Ob (Ob-T2D) rats with and without P-MSCs injection. (**e**,**f**) Bar diagram showing mean fold change in the glycolytic intermediate metabolites analyzed by LC–MS/MS analysis in the subcutaneous (**e**) and visceral (**f**) adipose tissues of WNIN/Control and WNIN/GR-Ob (Ob-T2D) rats with and without P-MSCs injection. (**g**,**h**) Western blot analysis for the indicated proteins isolated from the subcutaneous (**g**) and visceral (**h**) adipose tissues from WNIN/Control and WNIN/GR-Ob (Ob-T2D) rats with and without human P-MSCs injection. Compared to the respective total proteins, the relative levels of phospho-proteins are also shown next to the western blots in the form of bar diagram. Blots are representative of four independent experiments. Statistical analyses were performed between the control and the P-MSCs injected groups, using two-way ANOVA comparing the WNIN/Control and WNIN/GR-Ob (Ob-T2D) rats (*p < 0.05, **p < 0.01). Error bars represent one standard deviation from the mean. n = 6 rats per group.

Next, we asked whether the injection of P-MSCs restores the glucose homeostasis in the adipose tissues of WNIN/GR-Ob (Ob-T2D) rats. For this, we first fractionated the SVF to enrich the immature and naïve adipocytes from mature subcutaneous and visceral adipocytes^33^, and compared the respective glucose uptake capacity using 2-deoxy-2-[(7-nitro-2,1,3-benzoxadiazol-4-yl)amino]-d-glucose (2-NBDG) assay, as explained in the materials and methods. SVF from the subcutaneous and visceral adipocytes of WNIN/GR-Ob (Ob-T2D) had less glucose uptake when compared to the SVF isolated from the subcutaneous and visceral adipocytes of WNIN/Control rats (Fig. 4a,b). Strikingly, a similar injection of P-MSCs had restored the glucose uptake capacity of the SVF from adipocytes isolated from the WNIN/GR-Ob (Ob-T2D) rats and was comparable with the WNIN/Control rats (Fig. 4a,b). However, we did not observe any significant change in the glucose uptake with or without P-MSCs injection (Fig. 4a,b) in WNIN/Control rats' adipocytes.

Hexokinase (HK) and Pyruvate kinase (PK) are the two rate-limiting enzymes involved in the glycolysis pathway^34^, and  their altered regulation has been well documented in Obesity and T2D^35^. Here we show that P-MSCs injection was indeed functional and demonstrated an increase in HK and PK enzyme activity in the WNIN/GR-Ob (Ob-T2D) adipose tissues and was comparable with that of WNIN/Control rats (Fig. 4c,d).

To gain further insights into the metabolic flux, we next quantified the glycolytic pathway early intermediates using the LC–MS/MS analysis, both in the WNIN/Control and WNIN/GR-Ob (Ob-T2D) rats, with or without P-MSCs injection. The impetus from our earlier studies showed that WNIN/GR-Ob (Ob-T2D) rats show IR, associated with a lower glucose uptake capacity^24,25,29,36^. In similar lines, WNIN/GR-Ob (Ob-T2D) rats showed reduced glycolytic early intermediates, both in subcutaneous and visceral adipose tissues, compared to the WNIN/Control rats (Fig. 4e,f). Treatment with P-MSCs to WNIN/GR-Ob (Ob-T2D) rats resulted in elevated key glycolytic early intermediates, i.e., Glucose-6-phosphate, Fructose-6-phosphate, and Fructose-1-6-bis phosphate (Fig. 4e,f). Note that there was two to threefold higher pyruvate levels in the WNIN/GR-Ob (Ob-T2D) rats' adipose tissues than the WNIN/Control rats (Fig. 4e,f). We did not observe any alteration in the pyruvate levels with the P-MSCs injection (Fig. 4e,f). All these results indicate the beneficial effects of P-MSCs therapy to restore the normal glucose uptake capacity of subcutaneous and visceral adipocytes in WNIN/GR-Ob (Ob-T2D) rats.

### P-MSCs treatment upregulates the PI3K-Akt signaling in adipose tissues and restores the Glut4 expression in the WNIN/GR-Ob (Ob-T2D) rats

Since P-MSCs injection to the WNIN/GR-Ob (Ob-T2D) rats had reverted their glucose uptake and utilization, we next focused on understanding whether P-MSCs-mediated enhanced glucose uptake also utilizes the same pathway^37^. Immunoblotting analysis showed that P-MSCs also upregulated the IRS-1 Ser612 phosphorylation and Akt Thr308 phosphorylation in both subcutaneous and visceral adipose tissues (Fig. 4g,h). In humans, PI3 kinase consists of a p100 catalytic subunit and a p85 regulatory subunit encoded by two isoforms, namely p85α and p85β^38^. We observed that adipocytes from WNIN/GR-Ob (Ob-T2D) rats showed decreased steady-state expression levels of both the PI3K subunits (Fig. 4g,h). Interestingly, P-MSCs injection into WNIN/GR-Ob (Ob-T2D) rats effectively restored the protein expression of both p85α and p85β, comparable to WNIN/Control rats, both in subcutaneous and visceral adipocytes (Fig. 4g,h). More importantly, we observed that the P-MSCs treatment significantly upregulated the Glut4 protein expression in both the adipose depots in WNIN/GR-Ob (Ob-T2D) rats’ similar to WNIN/Control rats (Fig. 4g,h). These results indicate that enhanced PI3K-Akt signaling-mediated Glut4 upregulation with P-MSCs injection facilitates increased glucose uptake vis-à-vis insulin sensitivity, as well illustrated in adipose tissues WNIN/GR-Ob (Ob-T2D) rats.

### P-MSCs enhance the glucose uptake by the adipocytes under the obesogenic milieu—an in vitro approach

**Figure 5:**
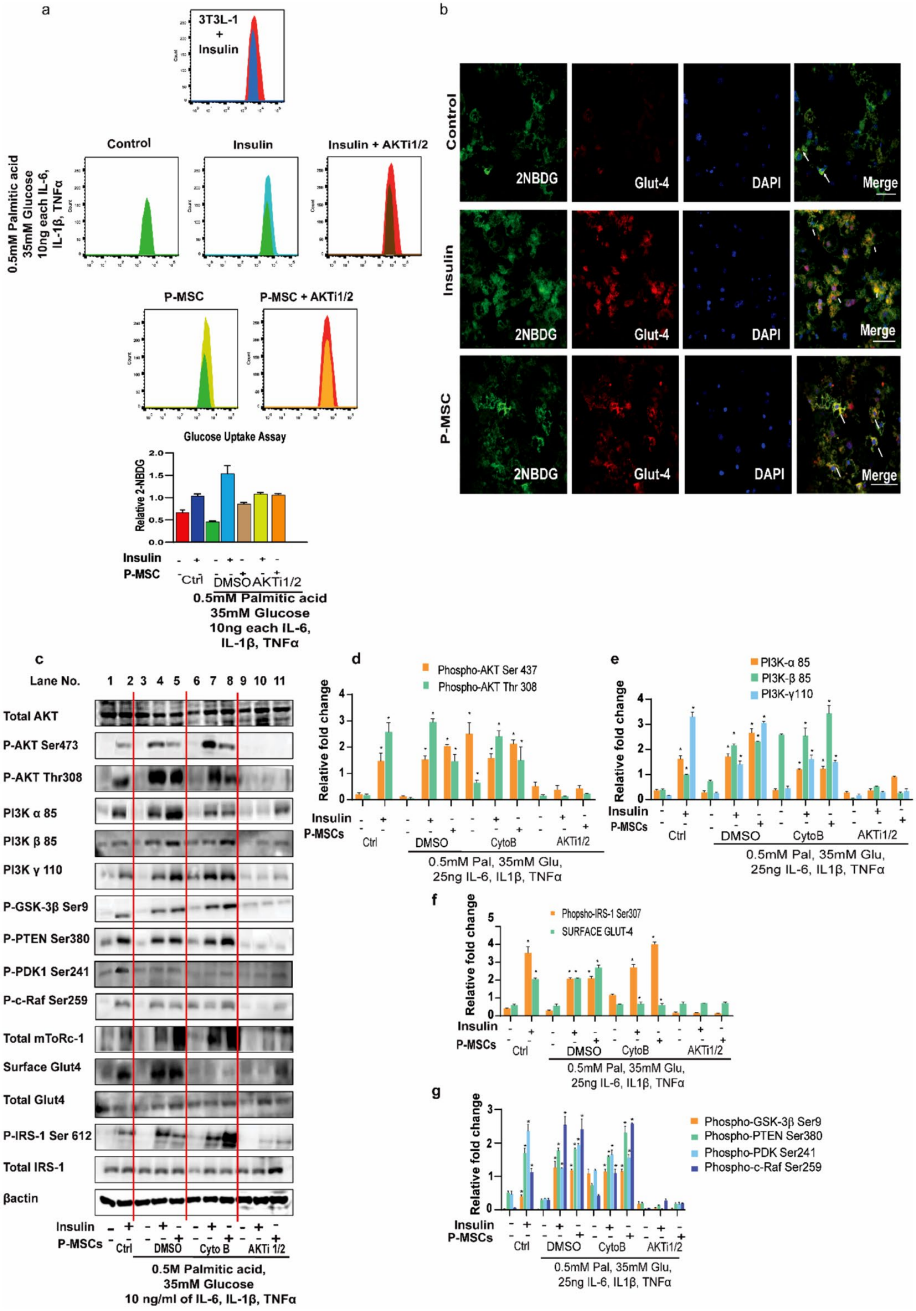
P-MSCs enhance the adipocyte glucose uptake and surface Glut4 translocation in adipocytes in a PI3K-AKT-dependent manner under obesogenic milieu. (**a**) FACS analysis of 3T3L-1 cells that are differentiated into adipocytes and co-cultured with human P-MSCs in the obesogenic milieu. Adipocytes were treated with AKT inhibitor (30 µM) for 30 min at the end of the co-culture. (**b**) Representative confocal images showing the co-localization of 2-NBDG and Glut4 in 3T3L-1 cells that were pre-differentiated into adipocytes and either stimulated with insulin (100 nM) for 20 min or co-cultured with human P-MSCs for 24 h. (**c**) Western blot images of the relative protein expression from the 3T3L-1 cells that were pre-differentiated into adipocytes and either stimulated with insulin or co-cultured with human P-MSCs, in the obesogenic milieu, as indicated. The cells were also treated with either AKT inhibitor (30 µM) for 30 min or with Cytochalasin B (25 µM) for 20 min to block the PI3K-Akt pathway or block the Glut4 surface translocation. (**d**–**g**) The relative levels of phospho-proteins and surface proteins for Glut4, compared to the respective total proteins, are also shown next to the western blots in the form of a bar diagram. Blots are representative of four independent experiments. Statistical analyses were performed between treated and untreated groups using two-way ANOVA (*p < 0.05, **p < 0.01). Error bars show one standard deviation from the mean.

To delineate the beneficial effects of P-MSCs treatment in the obesogenic milieu (Ob-T2D), we co-cultured P-MSCs with the adipocytes primed with and without high palmitate to induce obesogenic milieu. For this, we used 3T3L-1 cells, differentiated into adipocytes (with and without palmitate), and analyzed glucose uptake capacity by FACS analysis, using the 2-NBDG assay. Interestingly, we noted an enhanced uptake of glucose in adipocytes when co-cultured with the P-MSCs and in the presence of Insulin (Fig. 5a; compare the blue line with red line). The presence of high fat/obesogenic milieu had no significant impact on the glucose uptake capacity of adipocytes (Fig. 5a; compare blue with a green line), and insulin was also able to stimulate the glucose uptake under these conditions (Fig. 5a; compare blue with a green line). More importantly, P-MSCs co-culture had increased the glucose uptake capacity of these adipocytes in the obesogenic milieu (Fig. 5a; compare yellow with a green line). Note that this increased glucose uptake by the adipocytes is comparable to that of insulin stimulation. Since it is known that insulin stimulates glucose uptake via activating PI3K-Akt signaling, we presumed that P-MSCs also might be working through the same mechanism.

To elucidate the role of PI3K-Akt signaling in P-MSCs-induced glucose uptake by the adipocytes, we performed a similar assay as before, but in the presence of an Akt inhibitor. As expected, Akt inhibitor had decreased the insulin-stimulated glucose uptake capacity of adipocytes (Fig. 5a; compare red with brown line). More importantly, P-MSCs were unable to stimulate the adipocytes' glucose uptake capacity in the presence of AKT inhibitor (Fig. 5a; compare red with orange line). It is well known that PI3K- Akt signaling induces glucose uptake by glucose transporter 4 (Glut4) translocation into the membrane^39^. Further, the increased glucose uptake by the adipocytes in the presence of P-MSCs is due to enhanced Glut4 expression by the adipocytes, as seen by the immunofluorescence analysis (Fig. 5b). All these data suggest that P-MSCs activate the PI3K-Akt pathway in the adipocytes, which in turn leads to their enhanced glucose uptake.

### P-MSCs induce Glut4 surface translocation in adipocytes under obesogenic conditions in a PI3K-Akt-dependent mechanism

To confirm that P-MSCs indeed activate the PI3K-Akt signaling in adipocytes, we performed the immunoblot analysis under the same co-culture condition. Western blot analysis shows that insulin had stimulated the PI3K signaling, as demonstrated by Akt's increased phosphorylation and downstream targets, both in control and obesogenic conditions (Fig. 5c; compare lanes 1 with 2, and 3 with 4). Under the obesogenic conditions, P-MSCs co-culture had increased the Akt and its downstream signaling, similar to that of Insulin stimulation (Fig. 5c; compare lanes 5 with 3 and 4). Akt inhibitor had reduced the PI3K- Akt signaling activation induced by both Insulin (Fig. 5c; compare lane 10 with 9) and P-MSCs co-culture (Fig. 5c; compare lane 11 with 9). Irrespective of PI3K-Akt activation (either by insulin or by P-MSCs co-culture), we observed enhanced Glut4 surface translocation (Fig. 5c; compare lanes 1 with 2, 4, and 5 with 3). Akt inhibitor blocked the surface translocation of Glut4 (Fig. 5c; compare lanes 10, 11 with 9). Cytochalasin B is a well-known inhibitor for glucose transport^40^. Both Insulin and P-MSCs co-culture were able to enhance the surface translocation of Glut4 in control, but not in the cytochalasin B pre-treated cells (Fig. 5c; compare lanes 7 and 8 with 6).

## Discussion

The present study suggests for feasible application of P-MSCs therapy in the management of pre-clinical diabetes carried out in WNIN/GR-Ob (Ob-T2D) rats, which presents with Obesity, IGT, IR, and enhanced fat accumulation with age akin to human scenario^24,25^. In addition to becoming pre-diabetic, these rats also show impaired pancreatic β-cell functions and increasing obesity with age^25,36^. Interestingly, WAT accounts for 20 to 25 % of insulin-dependent glucose disposal in the body and plays a key role in maintaining glucose homeostasis^41^. In the present study, we have assessed glucose homeostasis, including insulin signaling, glucose sensitivity, and IR, in WATs before and after intramuscular injection of P-MSCs in WNIN/GR-Ob (Ob-T2D) rats. Our data reinstate the clinical efficacy of P-MSCs that intervened in WNIN/GR-Ob (Ob-T2D) rats to sensitize WAT to their endogenous insulin effectively, ameliorate glucose imbalance as well as normalize HOMA-IR, IGT, and ITT levels as compared to the Control rats (Fig. 2).

MSCs are one of the promising adult stem cells implicated in tissue repair^42^, and are well documented for their plasticity and differentiation potential into multiple cell types^43^. Their immunomodulatory and potent anti-inflammatory effects to blunt pro-inflammatory responses find their increased application in several inflammatory diseases^26,27^. P-MSCs have different secretory functions^44^, wherein paracrine secretion plays a major role in the therapeutic effects of the MSCs on inflammation. Previous reports have shown that MSCs constitutively express cytokines such as interleukin, IL-6, -7, -8, -11, -12, -14, -15, -27, leukemia inhibitory factor, macrophage colony-stimulating factor, VEGF expression, and stem cell factor in addition to IL-10 and TGF- β at the mRNA level^26,27^. Synergistic downregulation of pro-inflammatory cytokines and up-regulation of both pro-survival and anti-inflammatory factors advocate their therapeutic effects through autocrine and/or paracrine functions to modify the inflammatory environment^45^. Also, P-MSCs have been reported safe for transplantation therapy compared to other adult stem cells, due to their limited antigenic response^46^.

Multiple intramuscular injections (IM) of 1 × 10^6^ P-MSCs demonstrated more homing of fluorescent-labeled MSCs cells, predominant in the visceral region (Fig 2) of the adipose tissue of WNIN/GR-Ob (Ob-T2D) rats^24^, which correlates with the systemic insult of visceral adipose tissue, reported during the obese conditions^47^. MSCs mode of injection (intramuscular) into the thigh muscle also helps in the slow release of the paracrine secretion into the systemic circulation. The logic behind the intramuscular injection was to assess the cross-talk between the muscle and adipose tissues^48–50^. Several studies^51–53^ had shown that MSCs express multiple adhesion molecules and chemokine receptors, which aid in their migration towards various tissues in response to inflammation, and other major stimuli. In the present study, the adipose tissues exhibit a highly inflammatory microenvironment, wherein we postulate a similar migratory mechanism that could be suggestive of the homing pattern of the injected P-MSCs observed in the WNIN/GR-Ob (Ob-T2D) rats (Fig. 2a).

Among drug targets in treating obesity and allied complications, metformin appears most prominent and is the most common therapy, wherein it effectively sensitizes the muscle cells to Insulin^15,16^. Metformin also enhances the AMPK-mediated Glut4 membrane translocation in pre-adipocytes^54^. However, it has been shown that long-term metformin therapy exerts pro-inflammatory effects by inhibiting the NF-κB, through blocking of the PI3K-Akt pathway^55^. Metformin also causes secondary complications, resulting in the neuronal, kidney, and gastric malfunction^56^, paving the way for alternative therapies. Insulin stimulates the insulin receptor substrate-1 (IRS-1) phosphorylation, which leads to PI3K-Akt signaling activation^37^. Akt activation also enhances the expression and cell surface translocation of the Glut4 receptor^57^.

Interestingly, we have demonstrated that adipocytes isolated from subcutaneous and visceral adipose depots from WNIN/GR-Ob (Ob-T2D) rats had reduced Glut4 expression, probably underlining the low glucose uptake (Fig. 4). On the other hand, with P-MSCs therapy, we observed nearly a fourfold induction in the Glut4 expression in the WAT of WNIN/GR-Ob (Ob-T2D) rats, which correlates well with the enhanced glucose uptake of the SVF of WATs (Fig. 3). PI3K signaling is a central mechanism for performing key cellular functions, including proliferation, migration, and differentiation in addition to intracellular trafficking^37^, well exemplified in several systems^37^. We also observed that the P-MSCs therapy activated the PI3K-Akt signaling in the WATs (Fig. 4) and adipocytes (Fig. 5), suggesting the restoration of insulin signaling in these tissues (Fig. 4). Indeed, reduced Akt phosphorylation and its downstream effects due to inhibition of PI3K has been well documented in diabetes^37,58^, obesity^37^, as well as neurodegenerative diseases^37,58^.

To gain further insights into the metabolic functions of P-MSCs under the obesogenic milieu (Ob-T2D rats), we assessed its effects on glucose metabolism, which is regulated and orchestrated by several metabolic pathways^58^. It has also been reported that excess free fatty acids (FFAs) present in WAT under Ob-T2D conditions inhibit pyruvate dehydrogenase, impairing the muscle cells towards insulin-mediated glucose uptake and reducing glucose oxidation, which results in the accumulation of glycolytic metabolites in the muscle cells^59^. In the present study, with P-MSCs injection, we demonstrated an increase in glycolytic influx (Fig. 4), which was evidenced by a quantitative increase in the formation of glycolytic intermediates studied in the WATs of WNIN/GR-Ob (Ob-T2D) rats as compared to the controls. Our findings have been in agreement with several other reports that show impaired glycolysis under diabetic conditions^34,60^. Although P-MSCs therapy restored the early glycolytic intermediates in WNIN/GR-Ob (Ob-T2D) rats, pyruvate levels did not show any change with P-MSCs treatment and need to be explored further.

Inflammation is now accepted as a key factor in the pathology of T2D and possibly could be a key confounding player for β-cell death. Studies by Samuel et al.^59^ on the NLRP3 inflammasome pathway as critical indicators contributing to impaired adipose tissue dysfunction and pancreatic β-cells underlining pre-clinical diabetes. The high levels of FFAs present in such conditions also activate the NF-κB pathway, both of which lead to pro-inflammatory cytokine production^61^. Besides, there will be a decrease in the expression of the anti-inflammatory cytokines in the adipose tissues^62^. Our present results corroborate with these findings (Figs. 2f, 3a,b) and show that obesity with T2D depicts a state of chronic low-grade inflammation to impact the normal β-cell functions and leads to β-cell exhaustion and cell death^63^.

Cytokines play a crucial role in intracellular cell signaling and function in paracrine and endocrine signaling as immunomodulating agents, modifying the balance between humoral- and cell-mediated immunity^64,65^. The cytokines are known to attract the macrophages into the surrounding milieu, causing inflammation and IR in obese people^66^. Following published literature^64,65^, MSCs of perinatal origin (P-MSCs) have shown to be more effective to blunt the inflammatory response in target tissues en route via paracrine secretions of MSCs and their cytokines/interleukin profiles (IL-6, -7, -8, -11, -12, -14, -15, -27, LIF, M-CSF)^42^. In addition to IL-10 and TGF-β1, which may play a crucial role in negating pro-inflammatory responses, and attenuate inflammatory milieu underlining non-communicable diseases (NCDs)^67^. We also observed a baseline increase in the expression of pro-inflammatory cytokines in the WNIN/GR-Ob (Ob-T2D) rats, when compared to the control rats (Figs. 3 and 4); such as IL-6 (4.37-fold), IL-1β (4.125-fold), and TNF-α (2.55-fold) and a decreased expression of anti-inflammatory cytokines, such as IL-10 (ninefold), TGF-β (twofold), and IL-4 (eightfold).

Pro-inflammatory cytokines such as TNFα and IL6 are considered essential factors in developing obesity-associated IR^68^. In adipocytes, the IL-6 signaling pathway, and an increase in the expression of TNFα, IL-1β are thought to inhibit insulin signaling by reducing the insulin-stimulated phosphorylation of IRS-1, protein kinase B (PKB/Akt), which causes an increase in the basal FFA accumulation, resulting in lipid accumulation and IR^69^. Once these inflammatory signaling pathways are activated, they impair the insulin receptor and its mediated PI3K-PKB/Akt) activation of Glut4 translocation of glucose in adipose tissue.

MSCs therapy increases the surface Glut4 levels through activating the PI3K-Akt pathway^70^. MSCs are also shown to decrease the hypertrophy of adipocytes. However, the underlying pathways are not yet known^71^. Our findings in WNIN/GR-Ob (Ob-T2D) rats are in similar lines with pre-clinical diabetic subjects presenting with Obesity/IR. and IGT to show a nearly fourfold increase in the IL-6 expression^72,73^. IL-6 is generally derived from the SVF fraction of fat deposits, and its expression correlates directly to fat mass, i.e., with weight gain^74^. We observed that P-MSCs injection effectively ameliorated the obesogenic milieu in WNIN/GR-Ob (Ob-T2D) rats and significantly reduced the expression of IL-6, TNF-α, and pro-inflammatory cytokines (Fig. 3c,d), and upregulated the expression of anti-inflammatory cytokines. Though the paracrine mechanism appears most probable to explain the beneficial effects of P-MSCs, still the factors secreted by the MSCs and their beneficial effects need to be understood in situ.

Nevertheless, we show for the cytokine remodeling evidenced from the present results (Figs. 2 and 3) to benefit the suppression of inflammatory milieu as well as lessen the macrophage infiltration in the WAT (Fig. 3) probably a synergistic effect of these cellular events could perhaps contribute for the normalization of adipose functions with P-MSCs administration, which otherwise shows a high degree of expression in obesity as well as in T2D^75^. The anti-obesity effects of P-MSCs may be assigned to its paracrine and autocrine functions^28^ that are released from their perivascular location under tissue insult or injury^76^, or in modulating the niche and to activate  the resident stem cells^77^. In similar lines, the restoration of glucose homeostasis with P-MSCs intervention has been reported by Si et al. in diabetic model systems^70^.

Stimulation and activation of inflammation in obesity are multifactorial^78^. There is a need to study the role of glucotoxicity, FFAs, inflammasome stress kinases involved in obesity. This has been addressed in the present study by assessing the cross-talk between the high palmitate primed 3T3-L1 cells and co-culturing in vitro using a non-adherent system. We have tried to recapitulate the sequence of events to assess the glucose sensitivity and insulin signaling in-vitro to gain further insights into the beneficial effect of P-MSCs in the management of pre-clinical diabetes. We show that human placental MSCs co-cultured with 3T3-L1 cells induced PI3K-Akt pathway (Fig. 5) to increase insulin signaling protein phosphorylation, helping in glucose uptake and utilization.

Further, adipocytes (palmitate), when co-cultured with P-MSCs, show enhanced surface translocation of Glut4 into adipocytes (Fig. 5), which reconfirm our in vivo observations (Fig. 4). The addition of Glut4 inhibitor to the palmitate primed adipocytes inhibited this translocation, suggesting the specificity and regulation rendered by the P-MSCs towards glucose transportation^40^. Further, our data provide insights to show that activation of the PI3K-Akt pathway is required for Glut4 translocation onto the adipocyte membrane. We here envisage that during co-culture, the secretome that contains several growth factors, cytokines may be responsible for eliciting the beneficial effects appreciable in our present system.

**Figure 6:**
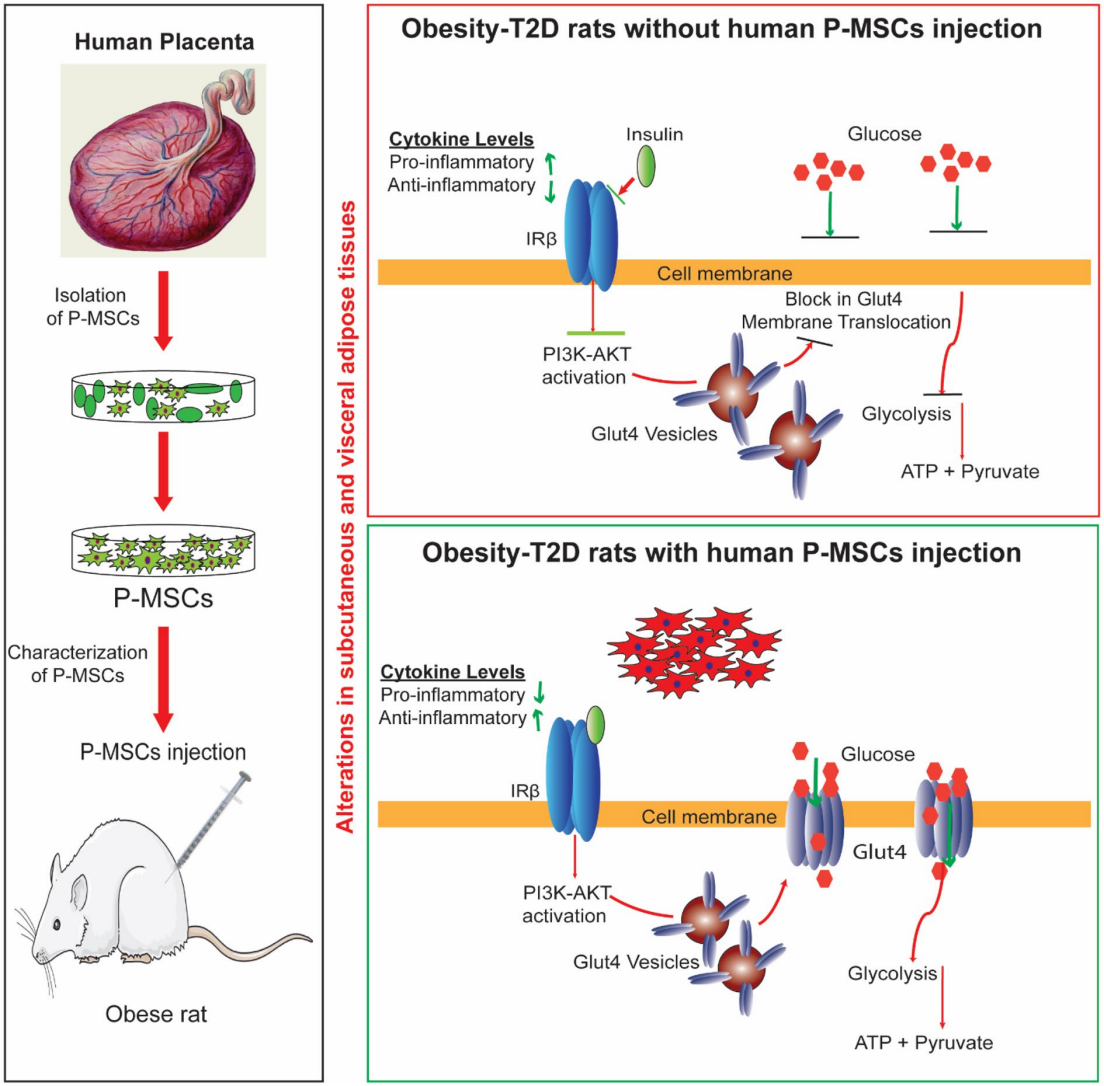
Schematic representation of Human Placental Mesenchymal Stem Cell Therapy in obesity-induced diabetes. Obesity-induced diabetes poses a major risk factor for the onset of metabolic syndrome with allied complications. White Adipose Tissues (WAT) show an altered dynamic of glucose and energy homeostasis under Ob-T2D conditions. P-MSCs therapy effectively re-establishes the dysregulated cytokines, activates the PI3K-Akt pathway, upregulates Glut4 expression, with enhanced glucose uptake and glycolysis in the WNIN/GR-Ob (Ob-T2D) rats.

Taken together, this study highlights the beneficial effects of P-MSCs administered in vivo and in vitro (co-culture) and highlights the dynamics across multiple stress simultaneously. Using co-culture, we have identified the role of insulin-stimulated and P-MSCs stimulated phosphorylation events in glucose transport which complement metabolite-driven effects in the coordinated glucose metabolism regulation (Fig. 6). We envisage that future investigations will delineate the interplay between these MSCs in regulating metabolism.

Overall, our findings from both in vivo and in vitro work show the promise(s) and potent applications of P-MSCs to negate IR, ameliorate IGT in the WAT, enhance glucose sensitivity, and blunt inflammation by remodeling the cytokine profiles and IGT. Our findings open up newer avenues and approaches of P-MSCs to address the clinical management of NCDs, including lipodystrophy patients.

## Materials and methods

### Isolation, expansion, and characterization of human P-MSCs

The chorionic plate of the placenta was exposed by stripping off the amnion. This chorionic plate was then washed with phosphate buffer saline pH 7.2 (PBS) to remove traces of cord blood. This chorionic plate of the placenta was subjected to 0.25% trypsin–EDTA digestion for 30 min at 37 °C. Isolation was carried out as previously described^79,80^. Their fibroblast morphology identified the human P-MSCs, and the cells were tested for viability, both Trypan Blue exclusion (TBE)^36^. Their multipotent functions at passage three, i.e., their ability to differentiate into chondrogenic, osteogenic, and adipogenic lineages, were assessed as per published protocols^22^. Adipogenic differentiation was briefly evaluated by adipogenic induction medium and adipogenic maintenance medium (Merck Millipore SCR020, USA), and adipogenesis was confirmed using Oil Red O Staining. For chondrogenic and osteogenic differentiation, 1 × 10^6^ P-MSCs/cm^2^ were plated onto tissue culture flasks, and chondrogenic, osteogenic lineages were induced by replacing the growth medium (DMEM/F12) with chondrogenic, osteogenic, differentiation bullet kit (C-28012, Sigma-Aldrich USA; SCR028, respectively, Merck Millipore, USA). Chondrogenesis was confirmed using Alcian Blue staining and osteogenesis by staining with Alizarin Red S. All the studies have been carried out using the third passage of P-MSCs, which has also been characterized for Stro-1 and CD133 cellular markers for P-MSCs.

### Animal model and study design

We confirm that all methods were carried out in accordance with relevant guidelines and regulations for using animals. Also, we confirm that all the animal studies were carried out in compliance with the ARRIVE guidelines. Six-month-old WNIN/Control and WNIN/GR-Ob (Ob-T2D) Rats from the inbred strain from ICMR—National Institute of Nutrition (NIN) were used (n = 24) for the experiments. The rats were divided into four groups, WNIN/Control (n = 6), WNIN/Control injected with P-MSCs (n = 6), WNIN/GR-Ob (Ob-T2D) rats (n = 6), and WNIN/GR-Ob (Ob-T2D) rats injected with P-MSCs (n = 6). All the experiments were done in duplicates, i.e., Three sets of experiments using 6 rats per group were used in this study. Experimental protocols were approved by the Institutional Animal Ethical Committee (IAEC Committee, NCLAS, NIN (ICMR). The biochemical, cellular, and molecular studies were conducted at the end of 7 weeks after the 3rd P-MSCs injection unless specified. P-MSCs/1 × 10^6^ suspended uniformly in 100 µl of 1× phosphate-buffered saline (1× PBS) were injected intramuscular/thigh region of rats, one-shot/per week × 3. The control rats received an equivalent of 100 μl of 1× PBS.

### P-MSCs tracking in vivo

Briefly, P-MSCs/third passage (1 × 10^6^) cells were stained with DiD, and homing of labeled cells was done similarly^81^. Briefly, the cells were incubated with DiD 10 µM (Life technologies, USA) for 1 h at 37 °C, using the manufacturer’s protocol, and suspended in 100 µl of 1× phosphate-buffered saline (1× PBS), and injected into the intramuscular/thigh region of rats, one-shot/per week × 3. In contrast, the controls received an equivalent amount of 1× PBS. At the end of 7 weeks of the experimental duration, the animals (3 animals/group) were measured for the longitudinal changes in fluorescent intensity at 640 nm (absorption) and 668 nm (excitation) every week post-injection using the IVIS Spectrum imaging system. The data were analyzed using Living Image software (PerkinElmer, USA).

### Glucose and insulin tolerance test (GTT/ITT)

At the end of the 7th week, we performed ITT and GTT after fasting the animals for 8–10 h. For GTT, the rats were infused with a 20% glucose solution (2 g of glucose/kg of body mass), i.p^82^. For ITT, we followed method^83^ of using the formula volume of IP glucose injection (μl) = 10 × body weight (g) and 0.75 IU of insulin/g body mass). The blood glucose levels were measured at 15, 30, 60, and 120 min after glucose and insulin injection. Glucose levels were measured using the ACCU-CHEK Advantage Meter (Roche Diagnostics GmbH, Mannheim, Germany).

### Enzyme-linked immunosorbent assay

Serum Insulin, C-peptide, and Glucagon concentrations were measured using specific ELISA kits (Mercodia, Sweden). The homeostatic model assessment (HOMA) described as previously^84^, was used to assess changes in IR (HOMA-IR) and pancreatic β-cell function (HOMA-β) in treated groups during the experimental period. The following equations were used to calculate the HOMA-IR index and HOMA-β index (HBCI): HOMA-IR index = (FBG [mmol/L] × FINS [units/L])/22.5) and HOMA-β = (20 × FINS [units/L])/(FBG [mmol/L] − 3.5).

### Measurement of serum and tissue cytokines (Dot Blot)

Serum and tissue cytokine profiling was performed according to the manufacture's protocol (Abcam, USA). Briefly, the tissue lysates and serum were incubated at 4 °C with the membrane overnight and washed with wash buffer I and II three times (5 min each) and the biotin-conjugated cytokines (CINC-2, CINC-3, CNTF, Fractalkine, GM-CSF, IFN-γ, IL-1α, IL-1β, IL-4, IL-6, IL-10, Leptin, LIX, MCP-1, MIP-3α, β-NGF, TIMP-1, TNF-α, VEGF) were incubated with a membrane overnight at 4 °C then washed wash buffer I and II for three times (5 min each), incubated with HRP-conjugated Streptavidin into each well and incubated for 2 h at RT, washed three times (5 min each) again, with wash buffer I and II before imaging with the ChemiDoc.

### Measurement of serum and tissue cytokine levels

The following major pro-and anti-inflammatory cytokines from serum and tissue lysates (visceral and subcutaneous) were analyzed: TNF-α, Monocyte Chemotactic Protein 1 (MCP-1; systemic name CCL2), IL-6, GM-CSF, IL-18, IFN-γ, Leptin, and VEGF were measured using multiplex map (Millipore, USA). IL-1β, IL-10, IL-12 p70, IL-13, IL-4, IP-10, TNFα, and TGF-β were analyzed by ELISA using the Procaratplex multiplex kit (Invitrogen, USA). All the multiplexing assays were performed as per the manufacturer's instructions.

### CD11b study by flow cytometer

Adipocytes have been isolated according to the previous protocols. Adipose tissue was subjected to collagenase  type I digestion for 1h at room temperature, passed through a 100 µ filtration sieve and spun at 1200 rpm for 5 min, and collected the SVF^85^. SVF was then treated with CD11b (Life Technologies, USA) at 10 µm/ml concentration for 35 min and washed with PBS, and analyzed further using a flow cytometer.

### Real-time quantitative PCR (cytokines markers)

The qRT-PCR analysis was performed as described previously^86^, with the following modifications. Total RNA was extracted using the Trizol reagent (DSS-Takara Biosciences, India), and cDNA synthesis was performed using the enhanced Avian Reverse Transcriptase (Sigma-Aldrich, USA) according to the manufacturer's instructions. The gene expression was analyzed using the 7500 Fast Real-Time PCR instrument (Applied Biosystems, Foster City, CA, USA). β-actin expression was used as the reference gene. The list of primers used for amplification is given in Supplementary Table-1 (qRT-PCR).

### Glucose transport assay

Glucose transport was assessed in the SVF using glucose analogue, 2-NBDG, as described previously^39^. At the end of 7 weeks, visceral and subcutaneous adipose tissues were dissected under sterile conditions and allowed to recover and processed in Krebs–Ringer HEPES (KRH) buffer containing 11.1 mM glucose for 1 h. After the initial recovery, the fat deposits were pre-treated with or without human recombinant insulin (100 nM) for 20 min in a shaking water bath. The isolation of SVFs was carried out using collagenase (type IV) digestion^39^ in the KRH buffer. After repeated washings in DMEM/F12 medium without glucose and filtration, the cells were diluted 5× times with Krebs–Ringer Phosphate (KRP) buffer pH 7.4, supplemented with 0.2% (w/v) BSA. Following this, 2-NBDG (0.5 mM) was added and incubated for 30 min at 37 °C in a shaking water bath. The cells were washed three times in ice-cold 1× PBS and kept on ice to prevent further insulin-stimulated membrane trafficking. 2-NBDG uptake was performed on ice to ensure that the uptake rate was solely dependent on the membrane trafficking that had occurred during the preceding period of insulin stimulation. A control sample, which lacks the 2-NBDG, was used to set the flow cytometer compensation and gate parameters. Each experiment was performed in duplicates, and the average was considered as one biological replicate.

### Glycolysis enzyme activity assays

The Hexokinase assay was performed as previously described^87^. Briefly, 20 µg of fresh tissue lysate was added to 1 ml of reaction buffer for hexokinase (50 mM Tris HCl (Sigma-Aldrich), pH 7.5, 10 mM MgCl_2_ (Sigma-Aldrich), 0.6 mM ATP (Sigma-Aldrich), 100 mM glucose (Sigma-Aldrich), 0.2 mM NADP+ (Sigma-Aldrich), and 0.1 units of glucose-6-phosphate dehydrogenase (Sigma-Aldrich)). Ten units of glyceraldehyde-3-phosphate dehydrogenase (Sigma-Aldrich) per ml was used for analyzing the Hexokinase activity. The Pyruvate kinase assay was performed as previously described^87^. Briefly, 20 µg of fresh tissue lysate was added to 1 ml of reaction buffer for pyruvate kinase (50 mM Tris HCl (Sigma-Aldrich), pH 7.5, 5 mM MgCl_2_ (Sigma-Aldrich), 5 mM ATP (Sigma-Aldrich), 0.2 mM NADH (Sigma-Aldrich) 100 mM KCl (Sigma-Aldrich), 5 mM Na_2_HPO_4_ (Sigma-Aldrich), 5 mM MgCl_2_ (Sigma-Aldrich), 0.01 mM AMP (Sigma-Aldrich)) 5 mM fructose-6-phosphate (Sigma-Aldrich), 5 units of triosephosphate isomerase (Sigma-Aldrich) per ml, 1 unit of aldolase (Sigma-Aldrich) per ml was added to check the pyruvate kinase activity. A negative and positive control has been included without tissue lysate and with 0.05 units of hexokinase and pyruvate kinase in both the assays. Enzyme activities were measured and represented as the change in absorbance/min, calculated using a linear portion of the obtained curve.

### Metabolomic analysis by LC–MS

Subcutaneous and Visceral adipose tissues were processed for metabolite analysis^40,88^. The samples were quickly snap-frozen with liquid nitrogen and incubated in – 80 °C for 10 min. The tissues were quickly ground and blended in a mortar. Powdered fat tissues (20 mg) were then placed into a 1.5 mL scaled Eppendorf tube containing 300 μl lysis buffer (9:1 methanol: chloroform), mixed with 500 μL water. The samples were immediately vortexed for 3 min, and the tissues were subjected to centrifugation at 16,000×*g* for 5 min at 4 °C to remove the cell debris. The supernatant was stored at − 80 °C, before proceeding for LC–MS/MS analysis. Glucose-1-Phosphate, Glucose-6-Phosphate, Fructose-1-Phosphate, Fructose-1,6-bisphosphate, Pyruvate, Phosphoenolpyruvate, and the standards were purchased from Sigma-Aldrich, USA. Chloramphenicol (internal standard) (Sigma-Aldrich, USA) was prepared and diluted to 100 ng/mL using methanol.

### Cell line and growth conditions

3T3-L1 fibroblasts were maintained as described previously^40^. Differentiation was induced at 100% confluence by adding 250 nM dexamethasone, 350 nM insulin, and 0.5 mM 3-isobutyl-1-methylxanthine for 72 h. At the end of time points, the cells were incubated in media containing 350 nM insulin for a further 72 h. The adipocytes were used between days 9 and 12 after initiation of differentiation for all the assays. For co-culturing, the 3T3-L1 were seeded in the lower chamber of trans-well plates and treated with TNF-α (10 ng/ml), IL-1β (10 ng/ml), IL-6 (10 ng/ml), and Palmitic acid (0.5 mM) and glucose (35 mM), after serum starvation for 48 h. At the end of 48 h, human P-MSCs were plated on the upper chamber of the trans-well plate using the same medium and co-cultured. The samples were collected 24 h after co-culturing and processed further. Akt inhibitor 1/2 was purchased from TCI Chemicals and used at 30 µM final concentration.

### Glucose transport assay

Glucose transport was assessed using glucose analogue, 2-NBDG, as previously described^39^, with the following modifications. 100 nM insulin was added for specified times, and to determine non-specific glucose uptake, 25 μM cytochalasin B was added 5 min before the addition of 2-deoxyglucose. A control sample lacking 2-NBDG was used to set the flow cytometer compensation and gate parameters for 2-NBDG positive and negative events.

### Immunofluorescence

Immunofluorescence experiments were carried out as described by us previously^36^. The cells were incubated with Glut4 primary antibody at 4 °C overnight. The following secondary antibody was used for visualization: goat anti-rabbit antibody conjugated with Cy3. The nuclei were counterstained with DAPI.

### Western blotting analysis (insulin signaling pathway)

Subcutaneous and visceral adipose tissue were processed for Western blotting^40,89^. The tissues were homogenized as described previously^90^, in 1×RIPA buffer containing 150 mM sodium chloride, 1% Nonidet P-40, 0.5% Sodium deoxycholate, 0.1% SDS (sodium dodecyl sulfate), 50 mM Tris (pH 8.0) and protease inhibitor (Pierce). Proteins were separated by SDS-PAGE, immunoblotted with indicated antibodies, and imaged by ChemiDoc. The tissue lysates (40 μg protein/lane) were briefly resolved by SDS-PAGE and transferred to PVDF membranes. Membranes were blocked for at least 1 h with 4% (w/v) skim milk powder in Tris-buffered saline with 0.1% Tween 20 (TBST). The membranes were incubated overnight at 4 °C with the following primary antibodies: anti-β-actin (1:1000, mouse monoclonal, pierce), anti-PI3K-α (1:1000, rabbit polyclonal, CST), anti- PI3K-β (1:1000, rabbit polyclonal, CST), anti-PI3K-γ (1:1000, rabbit polyclonal, CST), anti-AKT (1:1000, mouse monoclonal, CST), anti-P-Ser 473 AKT (1:1000, mouse monoclonal, CST), anti-P-Thr 308 AKT (1:1000, mouse monoclonal CST), anti-IRS-1 (1:1000, mouse monoclonal, CST), anti-P-Ser 307 IRS1 (1:1000, mouse monoclonal, CST), anti-Glut4 (1:1000, mouse monoclonal, CST), anti-P-c-Raf ser 259 (1:1000, mouse monoclonal, CST), anti-P-PTEN (1:1000, mouse monoclonal, CST), anti-P-PDK1 (1:1000, mouse monoclonal, CST), anti-mTOR (1:1000, rabbit polyclonal, CST) and anti-P-GSK3β (1:1000, mouse monoclonal, anti-IRS-1 (1:1000, mouse monoclonal, CST). Membranes were then washed three times, for 15 min with TBST, incubated for 1 h at room temperature with the respective secondary antibodies diluted 1/10,000 in 5% (w/v) skim milk powder in TBST. The membranes were rewashed three times, 15 min each with TBST, before imaging with the ChemiDoc using G-Box from Syngene international limited. All the gels/blots used in the pictures are in compliance with the digital image and integrity policies.

### Statistical analysis

The results represent the mean of 6 rats per group. The “p” values were calculated using the Two-way ANOVA for normally distributed data and the multiple comparison test. Statistical analysis was done using GraphPad Prism (Ver 8.0). Heatmaps were plotted using GraphPadprsim.

### Ethics approval

Institutional Animal Ethics Committees (IAECs): P35F/IAEC/NIN/11/2012/VV/WNIN. Institutional Human Ethics Committees: MHB/SCR/021.

## Supplementary Information

Supplementary Information.
