# Author Correction: The promise(s) of mesenchymal stem cell therapy in averting preclinical diabetes: lessons from in vivo and in vitro model systems

**DOI:** 10.1038/s41598-021-99380-z

**Published:** 2021-09-28

**Authors:** Nagasuryaprasad Kotikalapudi, Samuel Joshua Pragasam Sampath, Sinha Sukesh Narayan, Bhonde R., Harishankar Nemani, Sathish Kumar Mungamuri, Vijayalakshmi Venkatesan

**Affiliations:** 1 Division of Cell and Molecular Biology, ICMR-National Institute of Nutrition, Jamai‑Osmania P.O., Tarnaka, Hyderabad, 500007 India; 2 Division of Food Safety, ICMR-National Institute of Nutrition, Jamai‑Osmania P.O., Tarnaka, Hyderabad, 500007 India; 3 Department of Regenerative Medicine, Manipal Institute of Regenerative Medicine, GKVK Post, Bellary Road, Allalasandra, Yelahanka, Bangalore, 560065 India; 4 Division of Animal Facility, ICMR-National Institute of Nutrition, Jamai‑Osmania P.O., Tarnaka, Hyderabad, 500007 India; 5 Present Address: Dr. D. Y. Patil Vidyapeeth, Pune, 411018 India

Correction to: *Scientific Reports* 10.1038/s41598-021-96121-0, published online 20 August 2021

The original version of this Article contained an error in the spelling of the author Bhonde R. which was incorrectly given as Bhonde Ramesh R. The original Article and accompanying Supplementary Information file have been corrected.

