# Supplementary material for: The promise(s) of mesenchymal stem cell therapy in averting preclinical diabetes: lessons from in vivo and in vitro model systems
Source: Sci Rep. 2021 Aug 20;11:16983. doi: 10.1038/s41598-021-96121-0 (PMC8379204; doi:10.1038/s41598-021-96121-0)

**Supplementary Text – Kotikalapudi, Nagasuryaprasad, et al.**

**The promise(s) of mesenchymal stem cell therapy in averting preclinical diabetes –lessons from in vivo and in vitro model systems**

Nagasuryaprasad Kotikalapudi^1^, Samuel Joshua Pragasam Sampath^1^, Sinha Sukesh Narayan^2^, Bhonde R^3^, Harishankar Nemani^4^, Sathish Kumar Mungamuri^2^, Vijayalakshmi Venkatesan^1*^

**Supplementary Figure Legends:**


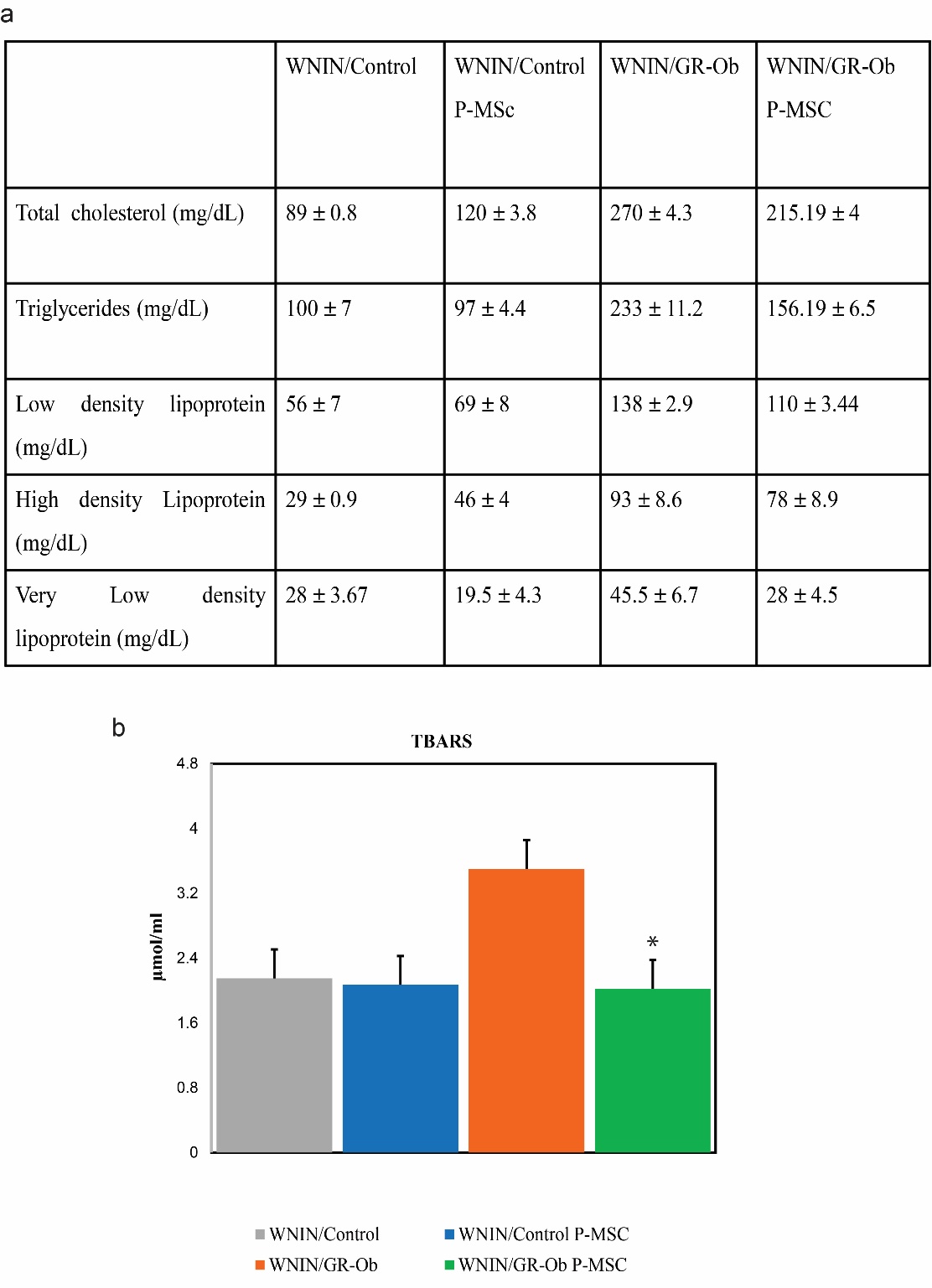


**Supplementary Figure.1: Human-PMSCs treatment restores the lipid profile and decreases lipid peroxidation. a)** Total cholesterol, LDL, HDL, VLDL levels in the blood samples collected from retro-orbital vein puncture before euthanization in WNIN/Control and WNIN/GR-Ob rats treated with or without human P-MSCs **b)** TBARS (p<0.05) indicative of the global oxidative stress/lipid peroxidation was observed in WNIN/Control, and WNIN/GR-Ob rats treated with or without human P-MSCs injection. n = 6 rats per group; *p < 0.05, **p < 0.01.


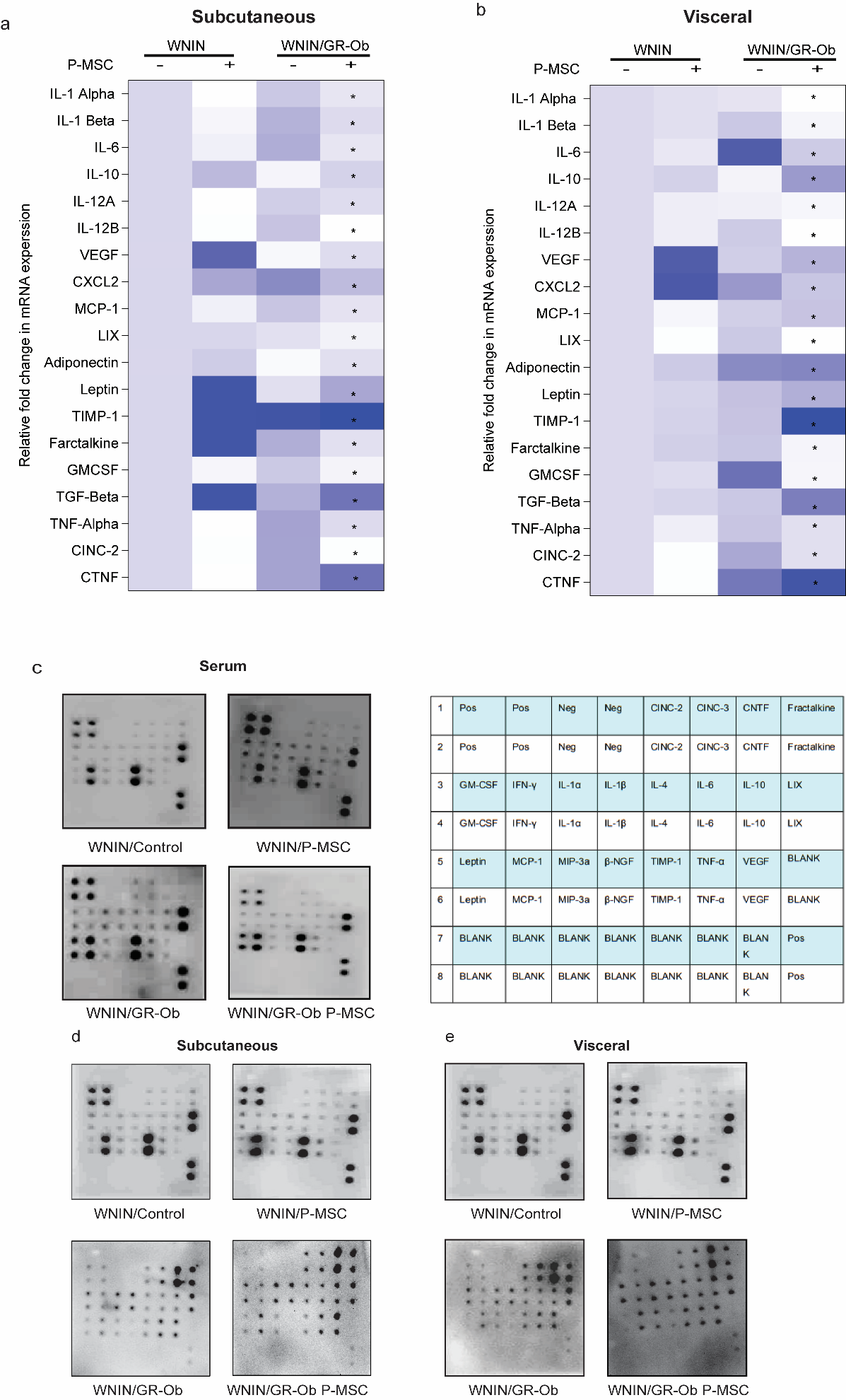


**Supplementary Figure 2: Human P- MSCs treatment restores the WNIN/GR-Ob rats cytokine expression. a, b)** Heat maps showing therelative mRNA expression levels of indicated cytokines in the subcutaneous (**a**) and visceral (**b**) adipose tissues of WNIN/Control and WNIN/GR-Ob rats with or without human P-MSCs treatment**, c, d)** Representative antibody array of the cytokinesreleased from the serum (**c**), subcutaneous (**d**), and visceral (**e**) adipose tissues of WNIN/Control and WNIN/GR-Ob rats treated with or without human P-MSCs injection.


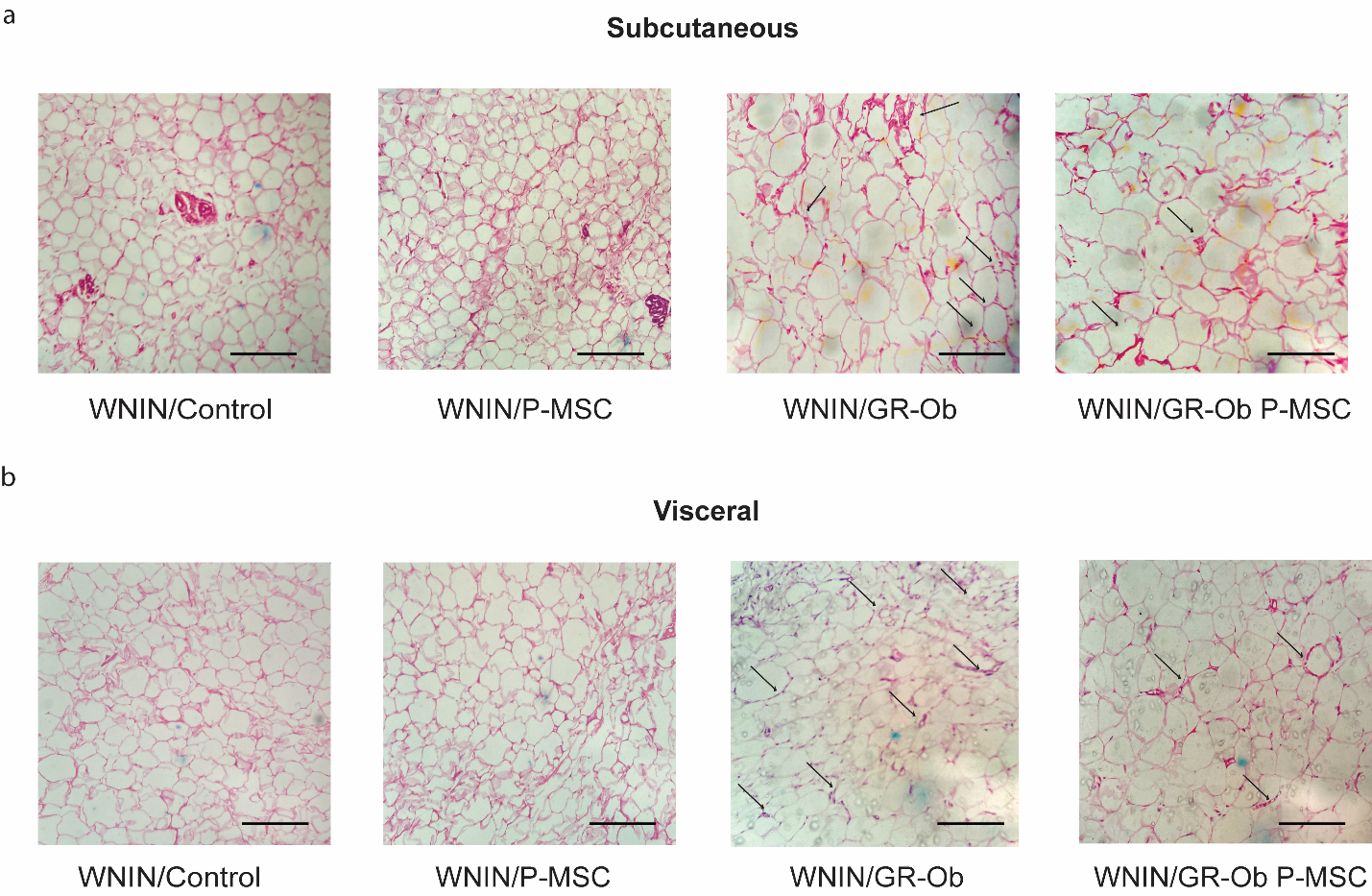


**Supplementary Figure 3: Human P- MSCs treatment reduces the WNIN/GR-Ob rats macrophage infiltration.** Representative images of H&E staining of subcutaneous (**a**) and visceral (**b**) adipose tissues of WNIN/Control and Ob-T2D rats with or without human P-MSCs treatment. Arrow indicates the infiltration of macrophages into the white adipocyte tissues. The bar indicates 100µM length.

**Supplementary Table 1:**

| RAT F Primer CXCL2 | AGGGTACAGGGGTTGTTGTG |
| --- | --- |
| RAT R Primer CXCL2 | TTTGGACGATCCTCTGAACC |
| RAT F Primer CINC-2 | CACTGCTTCTGCTGCTTCTG |
| RAT R Primer CINC-2 | TGACTTCTGTCTGGGTGCAG |
| RAT F Primer CTNF | CACCCCAACTGAAGGTGACT |
| RAT R Primer CTNF | ACCTTCAAGCCCCATAGCTT |
| RAT F Primer Fractalkine | CCAAGCAGAATGTTGGGTCT |
| RAT R Primer Fractalkine | GGCATGAATGGGTTCCTCTA |
| RAT F Primer IL-1α | GCAAAGCCTAGTGGAACCAG |
| RAT R Primer IL-1α | GCAGAAGGTGCACAGTGAGA |
| RAT F Primer IL-1 β | AGGCTTCCTTGTGCAAGTGT |
| RAT R Primer IL-β | TGAGTGACACTGCCTTCCTG |
| RAT F Primer IL-10 | GGGAAGCAACTGAAACTTCG |
| RAT R Primer IL-10 | GCTTTCGAGACTGGAAGTGG |
| RAT F Primer LIX | CGCTAATTTGGAGGTGATCC |
| RAT R Primer LIX | AGTGCATTCCGCTTTGTTTT |
| RAT F Primer Leptin | GAGACCTCCTCCATCTGCTG |
| RAT R Primer Leptin | CTCAGCATTCAGGGCTAAGG |
| RAT F Primer Adiponectin | AATCCTGCCCAGTCATGAAG |
| RAT R Primer Adiponectin | TCTCCAGGAGTGCCATCTCT |
| RAT F Primer TNF α | AGATGTGGAACTGGCAGAGG |
| RAT R Primer TNF α | CCCATTTGGGAACTTCTCCT |
| RAT F Primer IL-6 | CCGGAGAGGAGACTTCACAG |
| RAT R Primer IL-6 | ACAGTGCATCATCGCTGTTC |
| RAT F Primer Beta-actin | AGCCATGTACGTAGCCATCC |
| RAT R Primer Beta-actin | CTCTCAGCTGTGGTGGTGAA |
| RAT F Primer IL-12b | ACCCTCACCTGTGACAGTCC |
| RAT R Primer IL-12b | TTCTTGTGGAGCAGCAGATG |
| RAT F Primer IL-12a | AGCCATGTACGTAGCCATCC |
| RAT R Primer IL-12a | CTCTCAGCTGTGGTGGTGAA |
| RAT F Primer VEGF | GCCCATGAAGTGGTGAAGTT |
| RAT R Primer VEGF | ACTCCAGGGCTTCATCATTG |
| RAT F primer GM-CSF | TCCTAAATGACATGCGTGCT |
| RAT R Primer GM-CSF | GCCATTGAGTTTGGTGAGGT |
| RAT F Primer MCP-1 | ATGCAGTTAATGCCCCACTC |
| RAT R Primer MCP-1 | TTCCTTATTGGGGTCAGCAC |
| RAT F Primer TGF-β | GCAACTTGGAGGAGAACTGC |
| RAT R Primer TGF-β | GTCAGAGGCTCCAGGTCTTG |


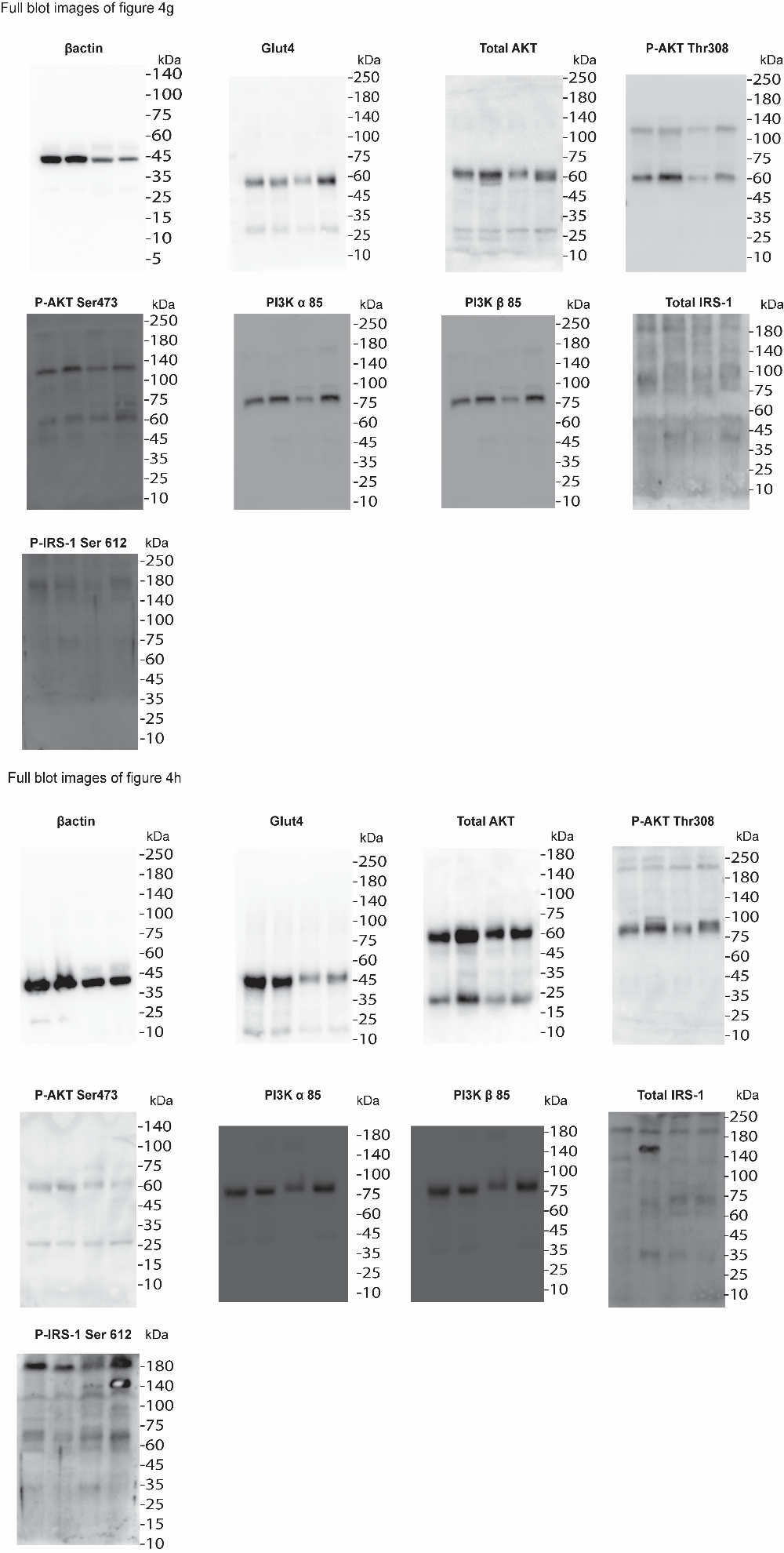


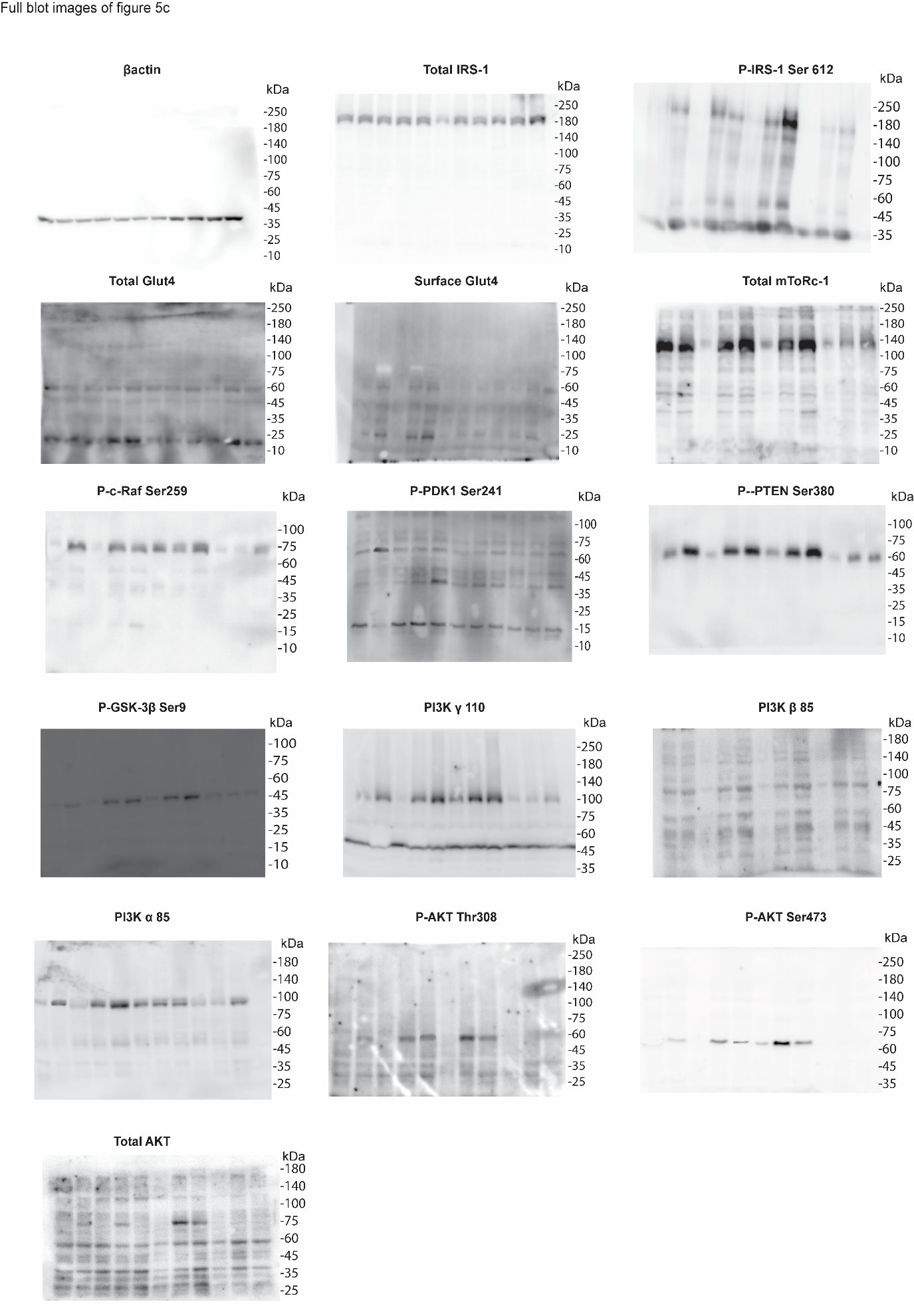

Supplement: Supplementary file 1 — Supplementary Information. [file 41598_2021_96121_MOESM1_ESM.docx]
